# Doxorubicin-induced loss of DNA topoisomerase II and DNMT1- dependent suppression of MiR-125b induces chemoresistance in ALK-positive cells

**DOI:** 10.18632/oncotarget.24465

**Published:** 2018-02-08

**Authors:** Annabelle Congras, Nina Caillet, Nouritza Torossian, Cathy Quelen, Camille Daugrois, Pierre Brousset, Laurence Lamant, Fabienne Meggetto, Coralie Hoareau-Aveilla

**Affiliations:** ^1^ Inserm, UMR1037 CRCT, F-31000 Toulouse, France; ^2^ Université Toulouse III-Paul Sabatier, UMR1037 CRCT, F-31000 Toulouse, France; ^3^ CNRS, ERL5294 CRCT, F-31000 Toulouse, France; ^4^ Institut Carnot Lymphome-CALYM, 31024, Toulouse, France; ^5^ Laboratoire d’Excellence Toulouse Cancer-TOUCAN, 31024, Toulouse, France; ^6^ European Research Initiative on ALK-related malignancies (ERIA) (http://www.erialcl.net/); ^7^ Equipe Labelisée LIGUE 2017

**Keywords:** miR-125b, chemoresistance, doxorubicin, DNA topoisomerase II, DNMT1

## Abstract

Systemic anaplastic large-cell lymphoma (ALCL) is a childhood T cell neoplasm defined by the presence or absence of translocations that lead to the ectopic expression of anaplastic lymphoma kinase (ALK), with nucleophosmin-ALK (NPM-ALK) fusions being the most common. Polychemotherapy involving doxorubicin is the standard first-line treatment but for the 25 to 35% of patients who relapse and develop resistance the prognosis remains poor. We studied the potential role of the microRNA miR-125b in the development of resistance to doxorubicin in NPM-ALK(+) ALCL. Our results show that miR-125b expression is repressed in NPM-ALK(+) cell lines and patient samples through hypermethylation of its promoter. NPM-ALK activity, in cooperation with DNA topoisomerase II (Topo II) and DNA methyltransferase 1 (DNMT1), is responsible for miR-125b repression through DNA hypermethylation. MiR-125b repression was reversed by the inhibition of DNMTs with decitabine or the inhibition of DNA topoisomerase II with either doxorubicin or etoposide. In NPM-ALK(+) cell lines, doxorubicin treatment led to an increase in miR-125b levels by inhibiting the binding of DNMT1 to the *MIR125B1* promoter and downregulating the pro-apoptotic miR-125b target BAK1. Reversal of miR-125b silencing, increased miR-125b levels and reduced BAK1 expression also led to a lower efficacy of doxorubicin, suggestive of a pharmacoresistance mechanism. In line with this, miR-125b repression and increased BAK1 expression correlated with early relapse in human NPM-ALK(+) ALCL primary biopsies. Collectively our findings suggest that miR-125b could be used to predict therapeutic outcome in NPM-ALK(+) ALCL.

## INTRODUCTION

Anaplastic large cell lymphoma (ALCL) is a biologically and clinically heterogeneous subtype of T cell lymphoma characterized by large lymphoid cells that express the Ki-1 (CD30) molecule [1]. Systemic ALCL are classified based on the presence or absence of the oncoprotein NPM-ALK, a fusion protein containing the amino terminal region of NPM1 (nucleophosmin1) juxtaposed to the entire intracytoplasmic domain of ALK (anaplastic lymphoma kinase) [2]. NPM-ALK(+) ALCL usually affects children and young adults, accounting for 10–15 percent of childhood lymphoma. Although long-term survival rates are currently high (85% of patients live longer than 5 years), approximately one in five patients relapses and tends to respond poorly to additional chemotherapy lines [3, 4]. Until now, no systematic analysis has been performed to search for molecular events that can explain the chemoresistance and/or early relapse observed.

In mediating tumorigenesis, NPM-ALK interacts with proteins involved in the regulation of cell proliferation, survival, motility and cytoskeletal rearrangements [5]. We and others have also highlighted the role of specific microRNAs (miRNA) in oncogenic ALK signaling in NPM-ALK(+) ALCL [6, 7, 8]. MiR-125b expression was low in mouse and various human NPM-ALK(+) lymphoma models, suggesting that miR-125b may play an important role in the pathogenesis of NPM-ALK(+) ALCL [9].

Mature miR-125b is transcribed from two loci located on chromosomes 11q23 (*MIR125B1*) and 21q21 (*MIR125B2*), and it is the human orthologue of lin-4, one of the very first miRNAs identified in *C. elegans* [10]. MiR-125b is ubiquitously expressed throughout the body and, like many miRNAs, its aberrant expression has been associated with different types of tumors where it is thought to act as either a tumor suppressor by down-regulating oncogene expression or a tumor promoter through the inhibition of tumor suppressor gene expression [11, 12, 13]. Despite its role in tumor development, the regulation of miR-125b expression is not well understood. Recent studies have shown that in solid tumors, the methylation state of CpG-rich regions correlates with the expression pattern of miR-125b. Hypermethylation has been associated with the silencing of tumor suppressor genes in several cancers and indeed patients with a hypermethylated miR-125b promoter region in solid tumors revealed shorter overall survival when compared to patients without a hypermethylated promoter [14, 15, 16]. This has led to the suggestion that low miR-125b expression could be a potential biomarker for clinical outcome. Indeed, methylated miRNAs are already showing great potential as diagnostic and prognostic biomarkers in cancer therapy [17], for example our laboratories reported that the levels of two miRNAs, miR-29a and miR-150 were reduced in NPM-ALK(+) ALCL cell lines and biopsy specimens as a consequence of DNA hypermethylation [8, 18].

It has been proposed that chemotherapy can exert a selective pressure on epigenetically-silenced drug sensitivity genes present in cell subpopulations, leading to acquired chemoresistance [19]. The anthracycline compound Adriamycin^®^ (generic name doxorubicin) is a hydroxyl derivative of daunorubicin and is one of the most common anticancer drugs [20], currently being used for the treatment of a broad spectrum of solid tumors, leukemias and lymphomas, including NPM-ALK(+) ALCL [21]. Polychemotherapy regimens containing doxorubicin are the standard first-line treatment for NPM-ALK(+) ALCL [3] and achieve high remission rates, however relapse and resistance occur in 25 to 35% of patients whose prognoses are invariably poor [14]. It has been shown that resistance to anthracyclins is promoted by repressive methylation [22], and in fact one of the means by which doxorubicin exerts its antitumor activity is by intercalating into DNA and impairing DNA topoisomerase II (Topo II) function which is needed for the proper maintenance of DNA methylation [23]. The DNA methylation enzyme DNA methyltransferase 1 (DNMT1) is the prototypical DNA methyltransferase and is widely assumed to be responsible for most of the methylation of the human genome, including the abnormal methylation found in cancers [24]. It is thought to be another target of doxorubicin since this enzyme is also required for doxorubicin-induced apoptosis [25]. In terms of drug resistance, miR-125b has been shown to increase cell resistance to doxorubicin and daunorubicin in leukemia and Ewing sarcoma by suppressing the expression of apoptotic mediators such as p53 and/or BAK1 (Bcl2 antagonist killer 1) [26, 27]. High BAK1 expression has been shown to correlate with drug sensitivity in malignant lymphopoietic cells whereas low BAK1 levels correlate with resistance and relapse [28]. We therefore investigated the role of miR-125b and BAK1 in the development of resistance to anthracyclins in NPM-ALK(+) ALCL, and the potential regulatory role of miR-125b promoter methylation in this process. Our results show that the level of miR-125b could be an important predictor for early relapse of NPM-ALK(+) ALCL following doxorubicin-containing polychemotherapy.

## RESULTS

### NPM-ALK acts as a driving force in miR-125b downregulation

**Figure 1:**
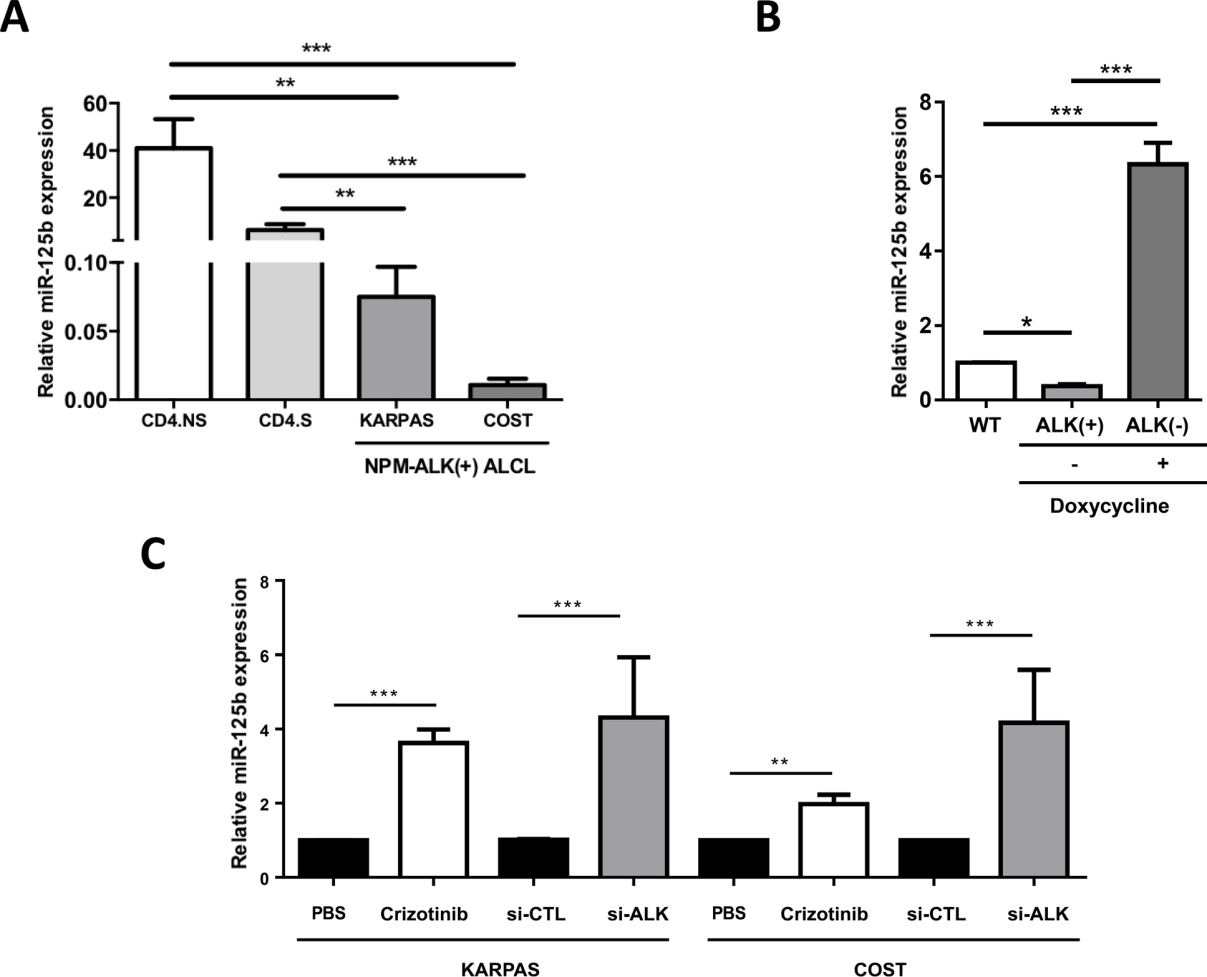
NPM-ALK represses the expression of miR-125b in ALCL human and mouse models (**A**) Quantitative real-time PCR (qRT-PCR) analysis of miR-125b expression in two NPM-ALK(+) ALCL cell lines (KARPAS-299 and COST) and CD4 lymphocytes stimulated (S) or not (NS) with CD3/CD28 antibodies. SNORD44 expression was used as an internal control. Relative human miR-125b expression was expressed as 2^–ΔCt^ relative to SNORD44 expression. (**B**) Assessment of miR-125b expression by qRT-PCR in wild type mice (WT, *N* = 6) or NPM-ALK transgenic mice containing a Tet-OFF system treated (+) or not (−) with doxycycline (*N* = 6; B). SNORD202 expression served as the internal control, and the relative ratio of mmu-miR-125b expression was expressed as 2^–ΔΔCt^ relative to WT mice. (**C**) MiR-125b expression in KARPAS-299 and COST cells treated for 72 hours or not (PBS) with crizotinib or transfected with either an irrelevant siRNA as the negative control (si-CTL) or a siRNA targeting ALK mRNA (si-ALK). SNORD44 expression served as the internal control and the relative ratio of hsa-miR-125b expression was expressed as 2^–ΔΔCt^ relative to untreated cells or to the si-CTL conditions. Data represent means ± SEM (bars) from 3 independent experiments. ^**^P < 0.001, and ^***^P < 0.0001; unpaired 2-tailed Student's *t* test.

Aberrant miR-125b expression has been demonstrated in a variety of hematological disorders [11, 12, 29, 30, 31], so we first investigated miR-125b expression in ALCL cells. Quantitative real-time PCR (qRT-PCR) showed that miR-125b expression was significantly lower in the NPM-ALK(+) ALCL cell lines KARPAS-299 and COST compared to non-tumorigenic CD4 lymphocytes both stimulated with CD3/CD28 antibodies (CD4.S, *N* = 3) or not (CD4.NS, *N* = 4) (Figure 1A). These results confirm those by Merkel et al. who reported a deregulation of miR-125b in NPM-ALK(+) cells [9]. To specifically investigate the role of the NPM-ALK oncoprotein, we used our previously published Tet-OFF conditional NPM-ALK lymphoma transgenic mouse model, where the removal of doxycycline from the animal feed induces NPM-ALK expression [32]. MiR-125b was significantly downregulated in lymph nodes isolated from mice with NPM-ALK(+) lymphoma (ALK(+), *N* = 6 for each treatment) (Figure 1B and Supplementary Figure 1A), compared with lymph nodes isolated from either control normal age–matched wild type littermate mice (WT, *N = 6*); or healthy transgenic mice who had received either doxycycline (*N* = 6) (Figure 1B) or the ALK inhibitor, crizotinib (*N* = 4) [ALK(−) (Supplementary Figure 1A)]. Both crizotinib and si-RNA directed against ALK mRNA (si-ALK) also induced a clear increase in miR-125b expression in KARPAS-299 and COST cells (Figure 1C). The efficient inhibition of ALK activity by crizotinib (Supplementary Figure 1B) and knockdown of NPM-ALK expression by si-ALK (Supplementary Figure 1C) was confirmed by western blotting. Together, these results suggest that the tyrosine kinase activity of ALK contributes to reduce miR-125b expression in ALK(+) models.

### DNA methylation is partly responsible for miR-125b silencing in NPM-ALK-positive ALCL cells

**Figure 2:**
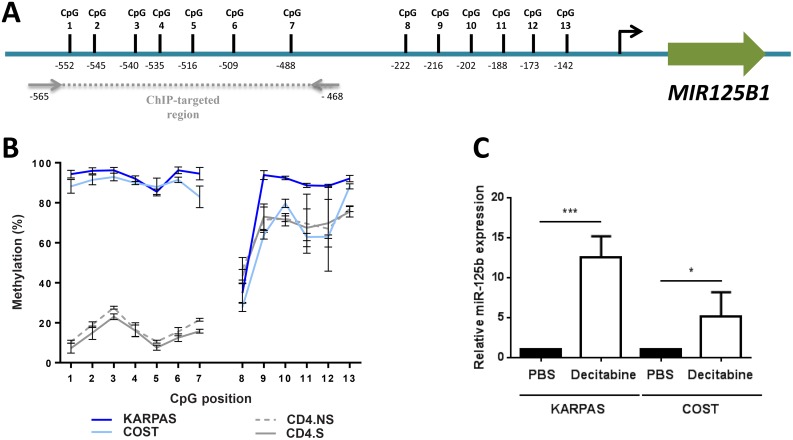
The *MIR125B1* promoter is methylated in NPM-ALK-positive ALCL cells (**A**) Schematic representation of the position of 13 CpG dinucleotides in the promoter region of *MIR125B1*. (**B**) Percentage of DNA methylation, assessed by bisulfite conversion and pyrosequencing in two ALCL NPM-ALK(+) cell lines, KARPAS-299 (KARPAS) and COST, and in CD4 lymphocytes stimulated (S) or not (NS) with CD3/CD28 antibodies. (**C**) Quantitative real-time PCR analysis of miR-125b expression in KARPAS and COST cells treated for 96 hours or not (PBS) with decitabine. SNORD44 was used as an internal control and the relative ratio of miR-125b expression was expressed as 2^–ΔΔCt^ relative to untreated cells. Data represent means ± SEM (bars) from 3 independent experiments, ^*^P < 0.05 and ^***^P < 0.0001; unpaired 2-tailed Student's *t* test.

The methylation state of CpG sites in the promoter region of miRNA genes is thought to correlate with their expression patterns in cancer [33], and hypermethylation of the *MIR125B1* promoter correlated with its reduced expression in solid cancers [14, 15, 16]. Thus, we carried out quantitative methylation pyrosequencing to assess the CpG methylation status of the −142 to −552 bp CpG-enriched region upstream of the *MIR125B1* gene, the area which is methylated in solid cancers (Figure 2A) [16]. We found that the CpG1 to CpG7 sites were significantly hypermethylated in NPM-ALK(+) ALCL cell lines compared to control CD4 lymphocytes (Figure 2B). Methylation levels varied from 76% to 100% in the NPM-ALK(+) cells with a mean of 93.55% and 89.25% in the KARPAS-299 and COST cells respectively, compared to 4% to 25% with a mean of 14.14% in the stimulated normal CD4/CD30(+) lymphocytes (CD4.S). No significant difference was observed between normal unstimulated CD4 (CD4.NS) and CD4/CD30(+) lymphocytes (CD4.S) (means of 17.28% and 14.14% respectively; Figure 2B). For the CpG8-CpG13 region, methylation levels were 43% to 83% with means of 63.39% and 63.48% for CD4/CD30(+) and unstimulated lymphocytes respectively (Figure 2B), and 27% to 97% with means of 82.29% and 64.75% in the KARPAS-299 and COST cells respectively (Figure 2B). Using normal CD4/CD30(+) lymphocytes as reference, methylation levels > 63% were taken to be hypermethylated, thus all of the CpG sites were considered hypermethylated in KARPAS-299 and COST cells (Figure 2B). We next analyzed the effect of decitabine, a DNMT inhibitor also referred to as 5-Aza-2′-deoxycytidine [34] (Supplementary Figure 2A). Decitabine significantly decreased methylation levels at these sites in NPM-ALK(+) ALCL cells, with mean ratios reduced from 94.29% (untreated) to 54.24% (decitabine) for KARPAS-299 and 87% to 49.07% for COST (Supplementary Figure 2A). Decitabine treatment was also found to increase miR-125b expression (Figure 2C). These data were confirmed in human NPM-ALK(+) primary tissues using lymph nodes taken from NPM-ALK(+) ALCL patients compared to normal reactive lymph node (RLN) samples (Supplementary Figure 2B). Methylation of the CpG1 to CpG7 sites was 24.04% and 14% in NPM-ALK(+) primary biopsies (*N* = 19) and RLNs (*N* = 10), respectively, indicating a slight increase in *MIR125B1* promoter methylation in NPM-ALK(+) samples, with a significant increase at CpG positions 3, 4 and 5 (*P* < 0.05) (Supplementary Figure 2B). Collectively, these data suggest that miR-125b silencing is due at least in part to aberrant *MIR125B1* promoter methylation in NPM-ALK(+) cells.

### BAK1 is a direct target of miR-125b in NPM-ALK-positive ALCL cells

**Figure 3:**
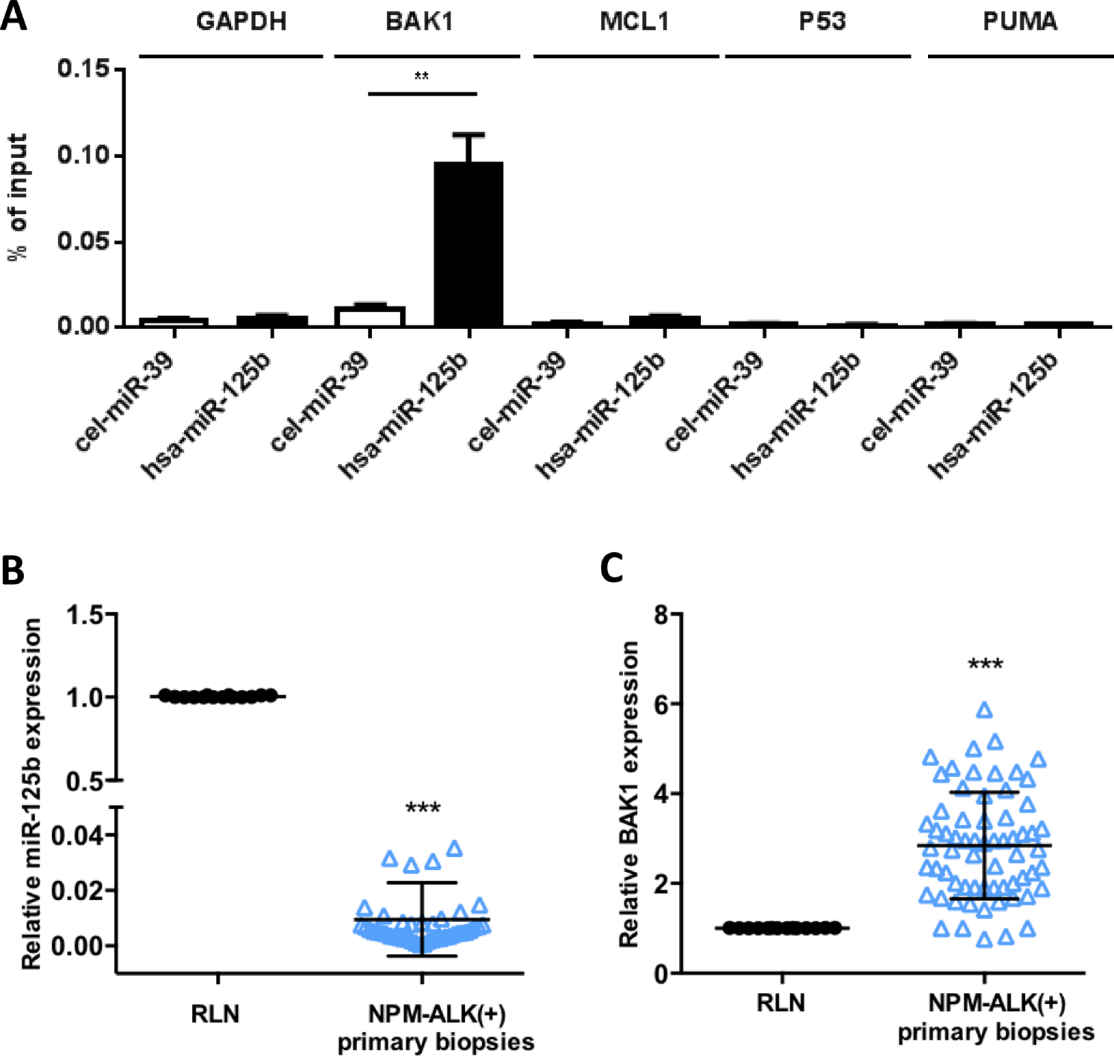
MiR-125b targets BAK1 mRNA in NPM-ALK-positive ALCL cells (**A**) NPM-ALK(+) ALCL KARPAS-299 cells were transfected with biotinylated forms of human miR-125b (hsa-miR125b) or *C. elegans* miR-39 (cel-miR39, an irrelevant microRNA, used as a negative control). Quantitative real-time PCR (qRT-PCR) analysis of BAK1, MCL1, PUMA, P53 or GAPDH mRNA was performed after pull-down of the biotinylated microRNAs using streptavidin beads. The results are presented as the percentage of input. (**B** and **C**) Quantitative RT-PCR analysis of (B) miR-125b expression relative to RNU1A and (C) BAK1 expression relative to GAPDH in reactive lymph nodes (RLN, *n* = 14) and NPM-ALK(+) primary biopsies (*n* = 65). RNU1A or GAPDH were used as internal controls and the relative ratios of miR125b or BAK1 expression were expressed as 2^–ΔΔCt^ relative to those in the reactive lymph nodes. Data represent means ± SD (bars); ^**^P < 0.001 and ^***^P < 0.0001; unpaired two-tailed Student's *t*-test with Welch's correction.

**Figure 4:**
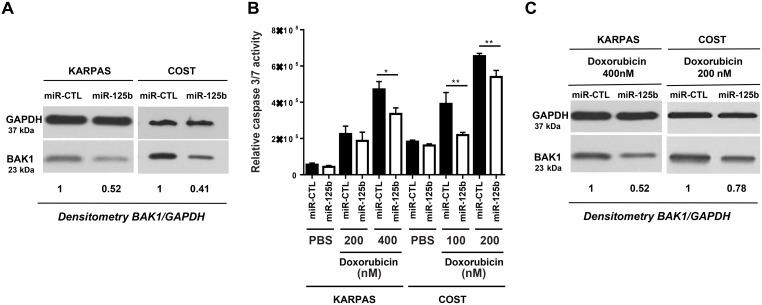
MiR-125b/BAK1 signaling impairs the doxorubicin response of NPM-ALK-positive ALCL cells In NPM-ALK(+) ALCL KARPAS-299 and COST cells transfected with a mimic miR-125b (miR-125b) or negative control microRNA (miR-CTL). (**A**) Western blotting analysis of BAK1 and GAPDH protein levels in cells transfected with a mimic miR-125b (miR-125b) or negative control microRNA (miR-CTL). Measurement of caspase 3/7 activity (**B**) and western blotting analyses of BAK1 and GAPDH protein levels (**C**) in cells treated with doxorubicin and transfected with either miR-CTL or miR-125b mimics. Densitometric analysis was performed using GeneTools software from Syngene. ^*^P < 0.05 and ^**^P < 0.001; unpaired 2-tailed Student's *t* test.

In several cancers, miR-125b induces chemoresistance to anthracyclins through suppression of various apoptotic modulators [26, 27, 35], thus in KARPAS-299 cells we evaluated whether miR-125b interacts with BAK1, P53, PUMA and MCL1 mRNAs which were identified as directly targeted by miR-125b [36, 37]. Biotinylated forms of human miR-125b (*hsa*-miR-125b) and *C. elegans* miR-39 (*cel*-miR39) (control) were used to co-purify any associated mRNAs. BAK1 mRNA co-purified with biotin-*hsa*-miR-125b at significant levels (*p* < 0.001) but not with the negative control (Figure 3A). In contrast, P53, PUMA, MCL1 and GAPDH mRNAs were not enriched in the biotin-hsa-miR-125b or the biotin-cel-miR-39 pull-downs (Figure 3A). Overexpression of miR-125b in KARPAS-299 and COST cells through transfection of an miR-125b mimic (miR-125b) or control scrambled microRNA (miR-CTL) induced a significant decrease in BAK1 protein (relative level 0.52 and 0.41 respectively, Figure 4A) and mRNA (*P* < 0.001) (Supplementary Figure 3A). BAK1 protein levels were also reduced by decitabine treatment in KARPAS-299 cells (relative level of 0.48 and 0.77 respectively, Supplementary Figure 3B), concomitant with the increase in miR-125b levels (Figure 2C). To confirm these results *in vivo*, lymph nodes samples from 65 NPM-ALK(+) lymphoma patients were analyzed using qRT-PCR (Figures 3B and 3C), selecting cases with at least 50% lymph node involvement. Despite the presence of a mixed population of neoplastic and normal cells, an inverse correlation between miR-125b (*P* < 0.0001) and BAK1 (*P* < 0.0001) mRNA expression was detected compared to RLN controls (*N* = 14). Together these results support a relationship between BAK1 and miR-125b and suggest that in NPM-ALK(+) cells BAK1 may be a functional downstream target of miR-125b.

### The reversal of miR-125b-silencing upon doxorubicin reduces BAK1 expression and induces doxorubicin resistance in NPM-ALK-positive ALCL cells

**Figure 5:**
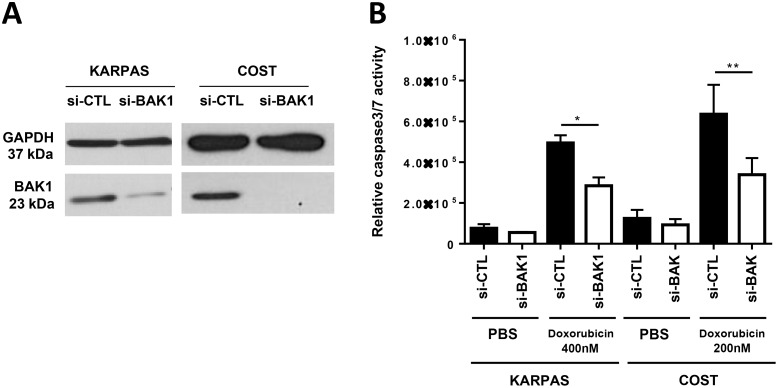
BAK1 silencing mimics the overexpression of miR-125b and leads to doxorubicin resistance in NPM-ALK-positive ALCL cells (**A**) Analysis in NPM-ALK(+) ALCL KARPAS-299 and COST cells of BAK1 and GAPDH expression by western blotting in cells transfected with an irrelevant siRNA as the negative control (si-CTL) or an siRNA targeting BAK1 mRNA (si-BAK1). (**B**) Assessment of caspase 3/7 activity in NPM-ALK(+) ALCL KARPAS-299 and COST cells transfected with si-CTL or si-BAK1 and treated for 48 h with 200 or 400 nM doxorubicin. Data represent means ± SEM (bars) from 3 independent experiments. ^*^P < 0.05 and ^**^P < 0.001; unpaired 2-tailed Student's *t* test.

We next investigated whether miR-125b overexpression and BAK1 repression affects the efficiency of doxorubicin in inducing apoptosis in ALCL cells. KARPAS-299 and COST cells were transfected with miR-125b or miR-CTL and then treated with either doxorubicin (concentrations of 100, 200 or 400 nM for 48 h) or vehicle (PBS). Caspase 3 activity was evaluated using the caspase-Glo^®^ 3/7 assay system in both miR-125b and miR-CTL-transfected KARPAS-299 and COST cells and was significantly reduced in cells transfected with miR-125b compared to miR-CTL-transfected cells (Figure 4B). We noted that overexpression of miR-125b in the absence of doxorubicin did not affect proliferation of NPM-ALK(+) cells (Supplementary Figure 5). To quantify the percentage of apoptotic cells after drug treatment the NPM-ALK(+) cells were stained with Annexin V-pacific blue/IP. As shown in Supplementary Figure 4, the rate of apoptotic cells (Annexin V-pacific blue positive cells) in miR-125b-transfected COST cells upon doxorubicin was statistically reduced compared with that of miR-CTL-transfected cells. Moreover, we observed that upon doxorubicin treatment cell viability was significantly enhanced in COST cells which ectopically expressed miR-125b (Supplementary Figure 5). It is to note that the successful overexpression of miR-125b in KARPAS-299 and COST, was confirmed by qPCR (Supplementary Figure 6). As previously reported by Panaretakis et al [37], we observed that doxorubicin treatment induces an increase of BAK1 expression (to a relative level of 1.78 and 1.91 for KARPAS-299 and COST respectively) (Supplementary Figure 7A). By contrast, the expression of BAK1 was inhibited when KARPAS-299 and COST cells upon doxorubicin were transfected with the mimic miR-125b (relative level 0.52 and 0.78 respectively, Figure 4C). Thus we considered whether an increase in miR-125b could induce NPM-ALK(+) cells to become resistant to chemotherapeutic agents through the downregulation of BAK1. Silencing of BAK1 expression in KARPAS-299 and COST cells using siRNAs directed against BAK1 mRNA (Figure 5A) clearly protected cells against doxorubicin-induced apoptosis, as assessed by a caspase-Glo^®^ 3/7 assay (Figure 5B). Taken together, these results suggest that miR-125b overexpression regulates pharmacoresistance in NPM-ALK(+) cancer cells by protecting against apoptosis through BAK1 suppression.

### DNA topoisomerase II and DNMT1 mediate miR-125b silencing in NPM-ALK-positive ALCL cells

**Figure 6:**
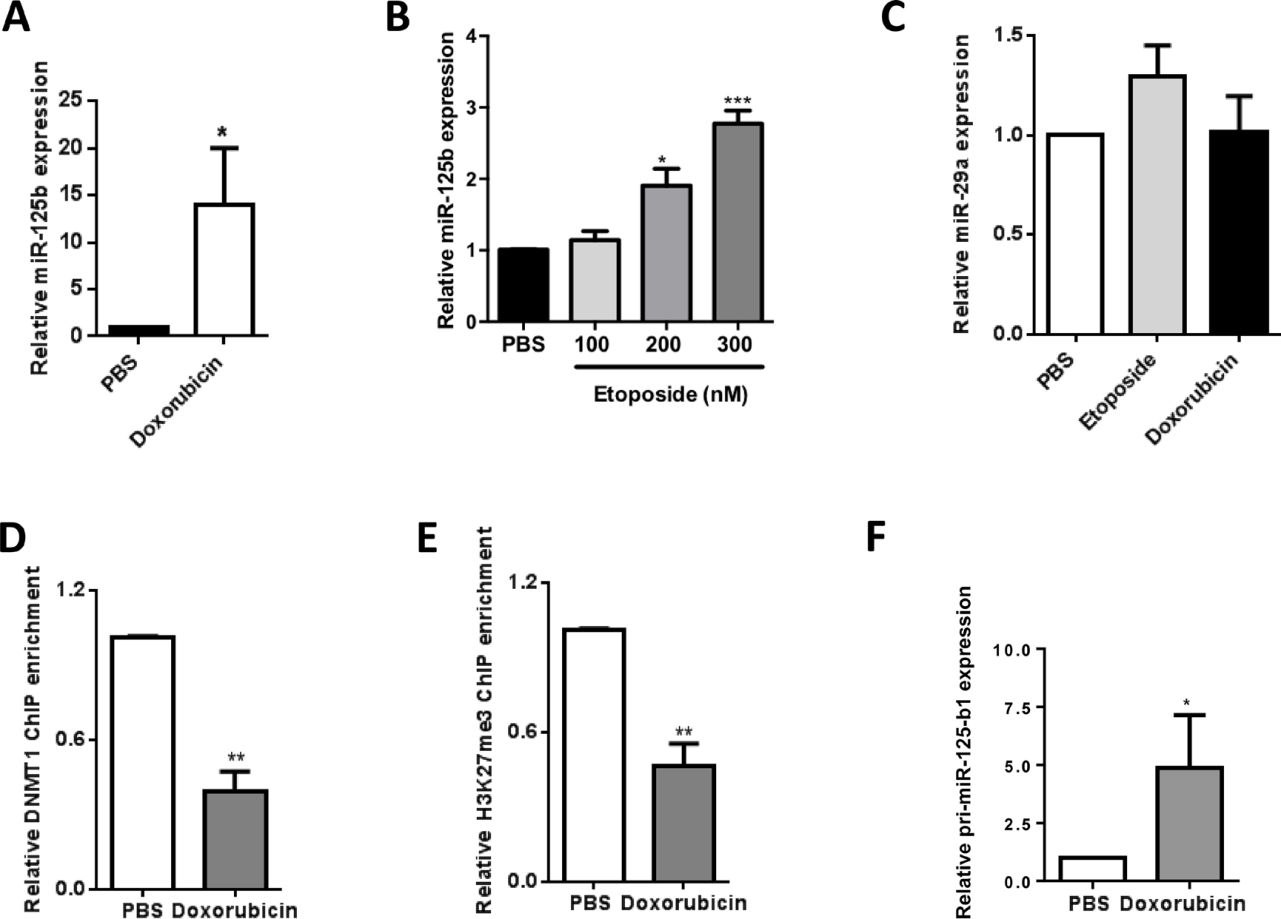
DNA methytransferase I and DNA topoisomerase II are mediators of miR-125b silencing in NPM-ALK-positive ALCL cells (**A** and **B**) Quantitative real-time PCR (qRT-PCR) analysis of miR-125b expression in NPM-ALK(+) ALCL KARPAS-299 cells treated for 48h or not (PBS) with 400 nM doxorubicin (A) or etoposide at a final concentration ranging from 100 to 300 nM (B). (**C**) Quantitative real-time PCR (qRT-PCR) analysis of miR-29a expression in NPM-ALK(+) ALCL KARPAS-299 cells treated for 48h or not (PBS) with doxorubicin or etoposide. SNORD44 served as an internal control and the level of hsa-miR-125b or miR-29a were expressed as 2^–ΔΔCt^ relative to untreated cells. (**D** and **E**) ChIP experiments were performed using antibodies against DNMT1 (D) or histone H3 trimethylated on Lys-27 (H3K27me3) (E) on KARPAS-299 cells treated for 48h or not (PBS) with doxorubicin. Results are expressed as a ChIP enrichment relative to the untreated condition (PBS). (**F**) qRT-PCR analysis of the expression of pri-miR125b1 in KARPAS-299 cells treated or not (PBS) with doxorubicin. Actin mRNA was used as the internal control and the relative ratio of pri-miR125b1 expression was expressed as 2^–ΔΔCt^ relative to untreated cells. (Data represent means ± SEM (bars) from 3 independent experiments. ^*^P < 0.05, ^**^P < 0.001, ^***^P < 0.0001; unpaired 2-tailed Student's *t* test.

Next, since we observed that doxorubicin treatment induced a clear increase in miR-125b expression in NPM-ALK(+) ALCL cells (Figure 6A), and since its major target is DNA topoisomerase II (Topo II), a DNA-binding protein required for the maintenance of DNA methylation by DNMT1 [22, 23], we assessed the role of Topo II in miR-125b regulation by treating cells with etoposide, a Topo II inhibitor that is part of the CHOEP (cyclophosphamide, doxorubicin, vincristine, etoposide and prednisone) chemotherapy regimen, at final concentrations ranging from 100 to 300 nM. Like doxorubicin, etoposide increased miR-125b expression (Figure 6B), showing that Topo II is involved in miR-125b regulation following doxorubicin treatment. By contrast, doxorubicin and etoposide did not affect miR-29a (Figure 6C), a miRNA down-regulated through DNA methylation in NPM-ALK(+) ALCL cell lines [3]. We next investigated whether the DNMT1 protein directly binds to the CpG-rich region upstream of the *MIR125B1* gene using chromatin immunoprecipitation (ChIP) experiments (Figure 2A). Inhibition of Topo II by doxorubicin markedly reduced DNMT1 binding to the *MIR125B1* promoter (Figure 6D) (*P* < 0.001). As previously reported by Yokochi et al [22], total DMNT1 protein levels were markedly reduced in doxorubicin-treated cells (Supplementary Figure 7A).

We also checked the cellular DNA methylation status of the CpG1 to CpG7 region upstream of the *MIR125B1* gene, which had been previously analyzed by ChIP (Supplementary Figure 7B), and found that this was not significantly altered by doxorubicin treatment in KARPAS-299 and COST cells. This lack of detectable demethylation despite the inhibition of DNMT by doxorubicin can be explained by the fact that the passive loss of DNA methylation requires several cell divisions to dilute out the methylated parental DNA strands. Similar results were previously reported by Yokochi et al. in a human colon carcinoma cell line [22].

In addition to its DNA methyltransferase activity, DNMT1 also represses transcription by cooperating with repressive histone modifiers [38]. In KARPAS-299 cells, ChIP analysis showed that doxorubicin reduced the levels of histone H3 trimethylated on its Lys-27 site (H3K27me3) that bound to the *MIR125B1* promoter (Figure 6E) (*P* < 0.001), an epigenetic mark that is associated with transcriptional repression [39, 40]. H3K27me3 was present at the *MIR125B1* promoter in NPM-ALK(+) cells not exposed to doxorubicin (Figure 6E), indicating the presence of non-permissive chromatin in this region. Since these data suggested that miR125b transcription was induced following doxorubicin treatment, we then evaluated pri-miR-125b1 expression in KARPAS-299 cells upon doxorubicin treatment using qRT-PCR and found them to be significantly higher (mean 4.86 ± 2.28, *P* < 0.05) relative to levels in untreated cells (Figure 6F). These results suggest that the loss of DNMT1 modulated chromatin structure.

We also considered the possibility that doxorubicin-induced upregulation of the *MIR125B1* gene was due to its capacity to directly intercalate into DNA and compete for the putative DNA binding site of transcription factors, such as Specificity protein 1 (Sp1) [41]. Sp1 binds to human DNMT1 [42] and to sites that are often embedded within CpG islands [43], such as those that are overrepresented in the *MIR125B1* promoter [44]. However, we observed no change in miR125b expression in NPM-ALK(+) ALCL cells following Sp1 inhibition by mithramycin A or si-RNA directed against Sp1 mRNA (data not shown). Taken together, these results suggest that both Topo II and DNMT1 inhibition contribute to turn on miR-125b expression in NPM-ALK(+) cells following doxorubicin exposure.

### MiR-125b downregulation is a biomarker for early relapse in human NPM-ALK-positive ALCL primary biopsies

**Figure 7:**
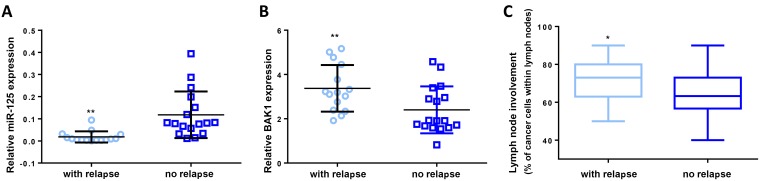
MiR-125b targets BAK1 mRNA in human NPM-ALK-positive ALCL primary biopsies Quantitative RT-PCR analysis of (**A**) miR-125b expression relative to *RNU1A* and (**B**) BAK1 expression relative to *GAPDH* in reactive lymph nodes (RLN, *N* = 14) and NPM-ALK(+) primary biopsies (*N* = 65). *RNU1A* or *GAPDH* were used as internal controls and the relative ratios of miR125b or BAK1 expression were expressed as 2^–ΔΔCt^ relative to those in the reactive lymph nodes. (**C**) Assessment by CD30 staining of frozen biopsies of percentage cancer cells in lymph nodes. Data represent means ± SD (bars); ^*^P < 0.05 and ^**^P < 0.001; unpaired two-tailed Student's *t*-test with Welch's correction.

Collegially our results suggested that, in NPM-ALK(+) cells, doxorubicin treatment could induce a miR-125b increase that allows the tumoral cells to become resistant to chemotherapeutic agent. Based on these results, we hypothesized that altered miR-125b expression in NPM-ALK(+) patients undergoing CHOP chemotherapy (which includes doxorubicin) could affect their response to treatment. Microarray microRNA-expression profiling was used to identify microRNAs differentially-expressed between NPM-ALK(+) patients (*N* = 52) and NPM-ALK(−) patients (*N* = 5) from ALCL samples obtained from chemotherapy-naive patients at diagnosis, normalized against equivalent miRNA levels from RLNs (*N* = 3) (Supplementary Tables 3 and 4). Venn diagrams from this data first identified 39 miRNAs, including miR-125b, that were significantly downregulated between NPM-ALK(+) and (−) primary tissues (Supplementary Figure 8), and also identified 6 miRNAs (including miR-125b) that were differentially expressed between NPM-ALK(+) primary biopsies of patients who had experienced early relapse (*N* = 28) and those without relapse after 3 years of minimal follow-up (*N* = 24) (Supplementary Figure 9, Supplementary Tables 5 and 6). qRT-PCR analysis of samples from the same NPM-ALK(+) cohort obtained from chemotherapy-naive patients at diagnosis showed a significantly lower abundance of miR-125b in patient who had experienced early relapse after CHOP chemotherapy (Figure 7A, *P* < 0.001). In addition, we observed a consistent downregulation of miR-125b and upregulation of BAK1 (Figure 7B, *P* < 0.001) in early relapsing (*N* = 15) compared to non-relapsing (*N* = 17) patients following normalization with miR-125b expression levels from RLNs (*N* = 14). Finally, we noted that early relapsing had a significantly higher histological nodal involvement compared to non-relapsing patients (Figure 7C, *P* < 0.05). Because after diagnosis, patients were all treated with CHOP chemotherapy, all together our data suggest that doxorubicin-containing chemotherapy could turn on miR-125b expression which might be linked to the recurrence of NPM-ALK(+) ALCL through BAK1 inhibition (Supplementary Figure 10), and therefore that alterations of miR-125b expression could predict therapeutic outcome.

## DISCUSSION

Our results show that NPM-ALK-mediated hypermethylation of the miR-125b promoter represses its expression and that miR-125b might influence sensitivity to chemotherapy in NPM-ALK(+) ALCL patients. These results are highly reminiscent of what we previously observed for two other microRNA genes, *MIR29A* and *MIR150*, whose levels were also reduced in NPM-ALK(+) ALCL as a consequence of promoter hypermethylation. MiR-29a and miR-150 were both shown to be tumor suppressors that, respectively, sensitized NPM-ALK(+) ALCL cells to doxorubicin-induced apoptosis through MCL-1 targeting, and reduced cell growth and viability through MYB [8, 18]. In the present work, functional characterization of miR-125b has revealed that it doesn't behave as a tumor suppressor but instead as a microRNA that mediates resistance to doxorubicin, a widely used chemotherapeutic drug. Indeed, miR-125b overexpression provided cytoprotection against doxorubicin-induced cell death through its capacity to target and inhibit BAK1 mRNA, a key pro-apoptotic factor crucial for the induction of doxorubicin-induced apoptosis in solid cancers [45]. Our findings may seem paradoxical since we show that a miRNAs that is not a tumor suppressor can be repressed by DNA hypermethylation, raising the question of whether selective repression of the *MIR125B1* gene in NPM-ALK(+) ALCL tumors is a biologically important event? One suggestion is that although miR-125b repression provides no growth advantage in the established NPM-ALK(+) ALCL cell lines KARPAS-299 and COST, it may have played a role during tumorigenesis. Another possibility is that hypermethylation of the *MIR125B1* gene could be a passenger DNA methylation with no effect per se on the tumorigenic process [46]. Nevertheless, our work has revealed that the upregulation of miR-125b by doxorubicin results in a negative feedback loop in which miR-125b reduces doxorubicin-induced apoptosis through the inhibition of BAK1 (Supplementary Figure 10). Our results show that the upregulation of miR-125b is most likely a consequence of the impairment of DNMT1 binding to the *MIR125B1* gene. This is probably due to both the decrease in DNMT1 protein levels seen upon doxorubicin treatment [22], and the reduction in DNMT1 recruitment to chromatin. Indeed, it has recently been shown that Topo II, a target of doxorubicin, is required for the maintenance of DNA methylation by DNMT1 [23]. We also cannot exclude the possibility that doxorubicin-induced upregulation of the *MIR125B1* gene is due to its capacity to directly intercalate into DNA and compete for the binding of some transcription factors [41], although we were able to rule out a role for Sp1 in this. Collectively, our findings suggest that the epigenetic profiling or expression analysis of miR-125b in NPM-ALK(+) ALCL could be used as a resistance marker/target to predict therapeutic outcome and improve the response of cells resistant to doxorubicin. This would also allow cases to be identified where doxorubicin could be combined with other cancer drugs to reduce its toxicity, for example doxorubicin-induced cardiomyopathy is an irreversible side effect and thus an important consideration in the treatment of curative malignancies in paediatric patients [47].

## MATERIALS AND METHODS

### Human cell lines, tumoral and normal samples

The human ALCL cell lines KARPAS-299 was obtained from DSMZ (German Collection of Microorganisms and Cell Culture) and COST was established in our laboratory. Cells were cultured in RPMI-1640 supplemented with 10% Fetal Calf Serum (FCS) and were maintained in exponential growth phase. CD4-positive T cells isolated from the peripheral blood of 3 healthy donors were used as normal lymphoid cells and were kindly provided by Dr Mary Poupot (UMR1037, CRCT). CD4-positive cells were stimulated for 2 days using CD3/CD28 antibodies coupled to magnetic beads (Dynabeads) in RPMI-1640 with 20% FCS. All cells were cultured at 37°C with 5% CO_2,_ and all media were supplemented with 2 mM L-glutamine, 1 mM sodium pyruvate, and 100 U/ml penicillin/streptomycin (all from Invitrogen).

Tumor samples from NPM-ALK(+) and NPM-ALK(−) ALCL were obtained after informed consent in accordance with the Declaration of Helsinki and stored at the “CRB Cancer des Hôpitaux de Toulouse” collection. The diagnosis of ALCL was based on morphological and immunophenotypical criteria, as described in the last WHO classification. Histopathological and immunostaining results were reviewed by a national (the French Lymphopath Network) or international panel of pathologists [3]. Only cases with at least 50% lymph node involvement (assessed by CD30 staining of frozen biopsies) and good RNA integrity were selected from our tumor bank. Antibody binding was detected with the Dako REAL Detection System (Code K5001). Lymph nodes collected from patients with reactive non-malignant disease, who were considered to be healthy donors, were retrieved from the CRB-Cancer du CHU de Bordeaux n° BRIF BB-0033-00036 – a member of the “Cancéropôle Grand Sud-Ouest” network.

### Cell treatment

ALCL cell lines were treated with 100 nM, 200 nM or 400 nM doxorubicin (Sigma-Aldrich) for 2 days and/or 2 μM decitabine (Sigma-Aldrich) for 2 to 4 days. NPM-ALK activity was inhibited using 500 nM crizotinib (@rtMolecule) for 3 days. Doxorubicin and crizotinib were added once to the cell culture and decitabine was then added every 2 days to the cell culture in order to maintain a constant concentration. Etoposide treatments were performed for 48 h at a final concentration of 100, 200 and 300 nM.

### Chromatin immunoprecipation

ChIP experiments were performed using the ChIP-it Express Kit provided by Active Motif. According to the manufacturer's instructions, the equivalent of 2.5 million cells were used for each immunoprecipation. The antibodies used are listed in Supplementary Table 2. Immunoprecipitated DNA and DNA for the input sample (20% of the starting material) were purified using the Chromatin IP DNA Purification Kit provided by Active motif. The purified-DNA samples were analyzed by qPCR (the primers used are listed in Supplementary Table 1) and the results are expressed as the percentage of input (100*2^[(Ctinput–[log2 of 5]) – CtChIP]).

### Purification of biotinylated microRNAs

Total cell lysates were prepared from KARPAS-299 cells 24 h post-transfection with biotinylated *hsa*-miR-125b-5p (*hsa*-miR-125b mercury LNA microRNA mimics premium, Exiqon) or biotinylated-cel-miR39 (negative control, Exiqon) in the following buffer (IP-buffer): 25 nM Tris-HCl pH 7.5, 200 nM NaCl, 0.2% Triton-X100, 5 mM MgAc, 1 mM DTT, protease inhibitors (Roche) and 0.2 U/μl RNase OUT (Invitrogen). Cell lysis was achieved by sonication. Streptavidin coupled to magnetic beads were first incubated with BSA (0.5 mg/ml) and yeast tRNA (0.2 mg/ml) for 30 min at 4°C and then washed twice with the IP buffer. Cell lysates were then incubated with the streptavidin beads for 2 h at 4°C. Beads were finally washed 5 times with 1 ml of IP buffer. RNAs were then extracted using the Trizol reagent (Invitrogen), according to the manufacturer's instructions.

### Statistics

Differences between two groups were examined using the unpaired 2-tailed Student's *t* test or unpaired 2-tailed Student's *t*-test with Welch's correction. All analyses were performed using GraphPad Prism version 6.00 for Windows. Differences were considered statistically significant when *P* < 0.05.

## SUPPLEMENTARY MATERIALS FIGURES AND TABLES








