# Supplementary material for: Doxorubicin-induced loss of DNA topoisomerase II and DNMT1- dependent suppression of MiR-125b induces chemoresistance in ALK-positive cells
Source: Oncotarget. 2018 Feb 8;9(18):14539–51. doi: 10.18632/oncotarget.24465 (PMC5865688; doi:10.18632/oncotarget.24465)
Supplement: Supplementary file 1 [file oncotarget-09-14539-s001.pdf]

Doxorubicin-induced loss of DNA topoisomerase II and DNMT1-dependent suppression of miR-125b induces chemoresistance in ALK-positive cells

SUPPLEMENTARY MATERIALS

SUPPLEMENTARY MATERIALS AND METHODS

RNA extraction, reverse transcription and quantitative PCR

Total RNA from cell lines and CD4-positive cells were extracted using the Trizol reagent (Ambion), according to the manufacturer's instructions. MiRNAs were reverse transcribed using the Universal cDNA Synthesis Kit II (Exiqon). The expression of miR-125b, RNU1A1 and SNORD44 (used as reference genes) was measured by quantitative PCR using a specific Exiqon PCR primer set (Supplementary Table 1) and a SYBR qPCR Premix Ex Taq (Tli RNaseH Plus) Kit from Takara. Messenger RNAs (mRNAs) were reverse transcribed using a SuperScript II Reverse Transcriptase (Invitrogen), according to the manufacturer's recommendations. Data are presented as relative quantities of target RNA. The high-throughput qPCR analysis method used for measuring BAK1 expression was performed using the primers listed in Table S1 and the BioMark 96 × 96 gene expression platform, according to the manufacturer's instructions (Fluidigm).

MiRNA and siRNA transfections

0.5 nmol of si-RNAs or microRNA mimics (miR-125b-5p mimic microRNA, mirVana hsa-miR125 MC10148, and negative control mimic mirVana control AM17110; Thermo Fisher) were electro-transfected using the Biorad Gene pulser (at 250 V and 950 µF) into 5.10^6 ALCL cells resuspended in 400 µl of serum-free medium. Immediately after transfection cells were transferred into 5 ml pre-warmed complete RPMI-1640 medium. The si-RNA sequences are listed in Supplementary Table 1.

MiRNA expression microarray

Microarray experiments were carried out using 1-color hybridizations on a human miRNA microarray (V3, 8 × 15K) (Agilent, Cat No: G4471A-021827): one glass slide formatted with eight high-definition 15 K arrays, based on the Sanger miRbase (release 12.0), with 866 human and 89 human viral miRNA probes represented. The mean normalized signal from the biological replicates was used for comparative expression analysis. A paired *t*-test with Benjamini-Hochberg correction (P value ≤ 0.05) was used to identify miRNAs differentially-expressed between tumor and benign tissues. The fold change in expression between tumor and benign samples was calculated from the normalized values.

DNA bisulfite treatment and bisulfite pyrosequencing

Genomic DNA (gDNA) was extracted using either the QIAamp DNA Mini Kit (Qiagen) or the Allprep DNA/RNA/miRNA Kit (Qiagen), according to the manufacturer's instructions. Bisulfite conversion was then conducted with 500 ng gDNA, using the MethylEdge Bisulfite Conversion System (Promega). Two regions of interest in the miR125-b promoters, containing 7 and 6 CpGs respectively, were amplified by PCR (PCR PyroMark Kit, Qiagen), with specific primers designed by the PyroMark Assay Design software (Qiagen), one of which was biotinylated for subsequent binding of the PCR products to streptavidin-coated sepharose beads. Methylation analysis was performed on the PyroMark Q24 Pyrosequencing System (Qiagen) using specific sequencing primers (Supplementary Table 1) and products and protocols supplied by the manufacturer.

Protein extraction and Western blotting analyses

Total cell lysates were prepared in RIPA buffer (20 mM Tris-HCl, 150 mM NaCl, 4 mM EDTA, 0.5% Triton X100 and 0.2% SDS). Protein concentrations were determined using a Bradford assay (Biorad). The primary antibodies used are listed in Supplementary Table 2. HRP-conjugated anti-mouse (Jackson Immuno Research) or anti-rabbit antibodies (Cell Signaling) were used as secondary antibodies. Densitometric analysis was performed using GeneTools software from Syngene.

Cell proliferation assay

The CellTiter 96AQueus One Solution provided by Promega was used to assess cell proliferation/survival, according to the manufacturer's instructions.

Apoptosis luminescent assay

The Caspase-Glo® 3/7 Assay Kit provided by Promega was used to assess cell apoptosis, according to the manufacturer's instructions.

Annexin V/propidium iodide (PI) double staining assay

Cells were harvested and incubated with recombinant annexin V–pacific blue at a final concentration of 1 g/ml for 5 min at room temperature. Just before analysis by flow cytometry, PI was added to all samples to a final concentration of 10 g/ml. The percentage of cells annexin V positive is indicated.

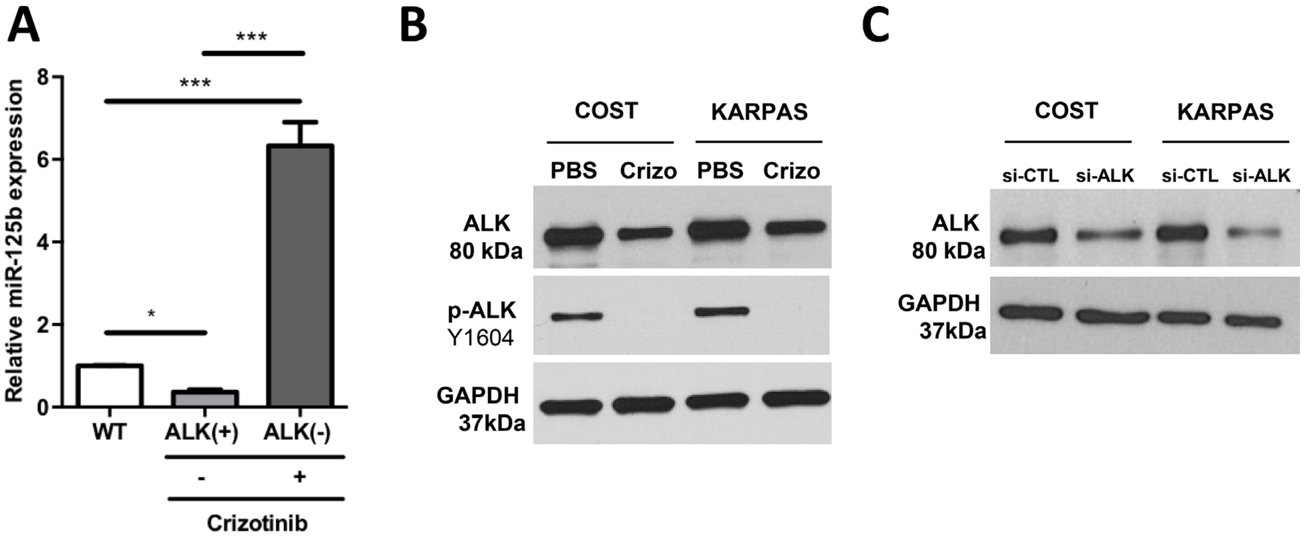

Supplementary Figure 1: NPM-ALK represses the expression of miR-125b in ALCL human and mouse models. (**A**) Assessment of miR-125b expression by qRT-PCR in wild type mice (WT, *n* = 6) or NPM-ALK transgenic mice containing a Tet-OFF system treated (+) or not (-) with crizotinib (*n* = 4). SNORD202 expression served as the internal control, and the relative ratio of mmu-miR-125b expression was expressed as $2^{-\Delta\Delta Ct}$ relative to WT mice. (**B** and **C**) Protein levels of NPM-ALK, p-NPM-ALK and GAPDH were assessed by western blotting in KARPAS-299 and COST cells treated or not (PBS) with crizotinib (Crizo) (B) or transfected with either an irrelevant siRNA as the negative control (si-CTL) or an siRNA targeting ALK mRNA (si-ALK) (C). Data represent means ± SEM (bars) from 3 independent experiments. $^{*}P < 0.05$ and $^{***}P < 0.0001$; unpaired 2-tailed Student's *t* test.

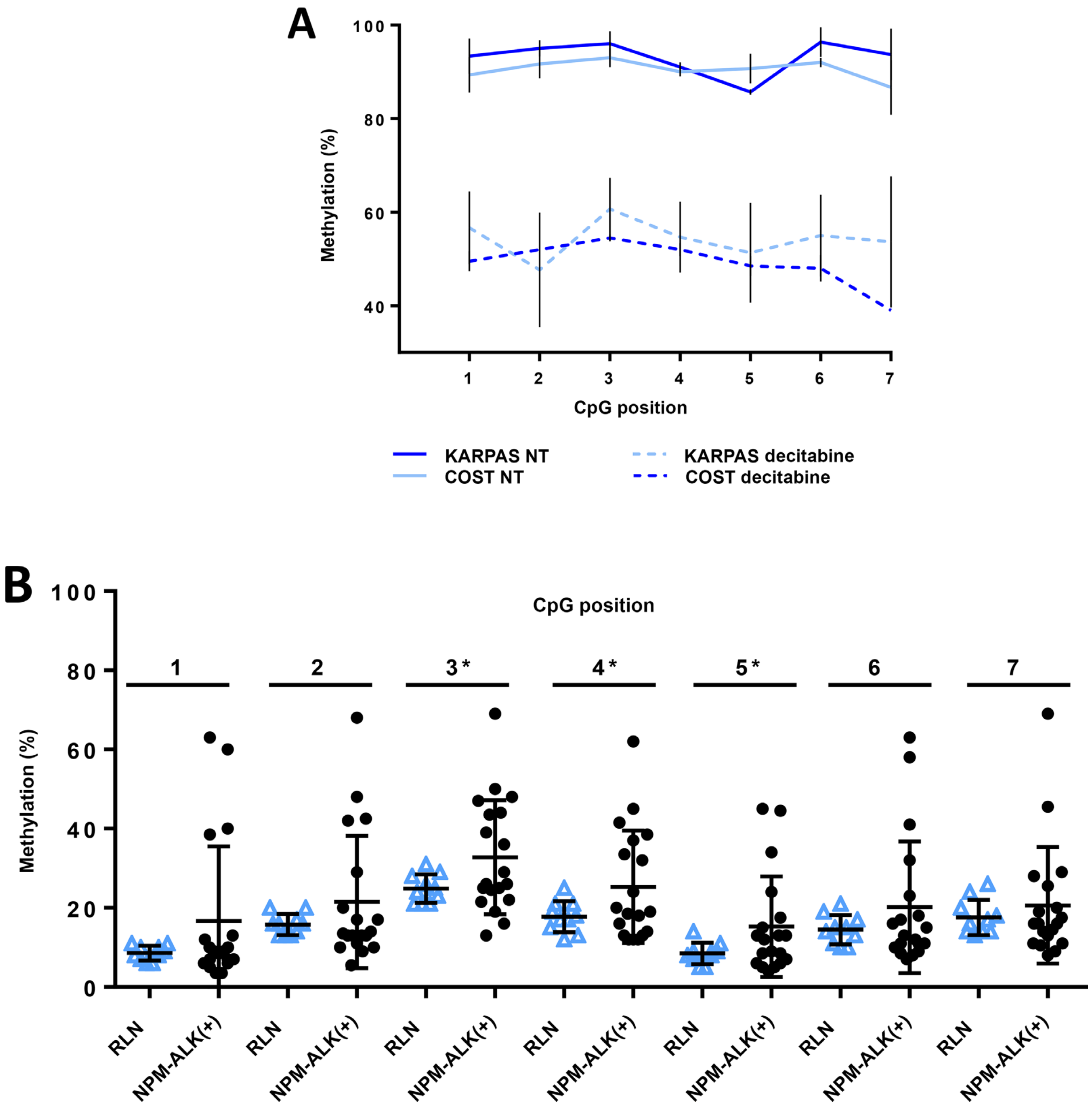

Supplementary Figure 2: The *MIR125B1* promoter is methylated in NPM-ALK-positive ALCL cells. (**A** and **B**) Percentage of DNA methylation, assessed by bisulfite conversion and pyrosequencing in KARPAS-299 (KARPAS) and COST cells treated or not (NT) with decitabine (A) and (B) in reactive lymph nodes (RLN; n = 10) and NPM-ALK(+) primary biopsies [NPM-ALK(+), n = 19]. Data represent means ± SD (bars); $^{*}p < 0.05$, unpaired two-tailed Student's t-test with Welch's correction.

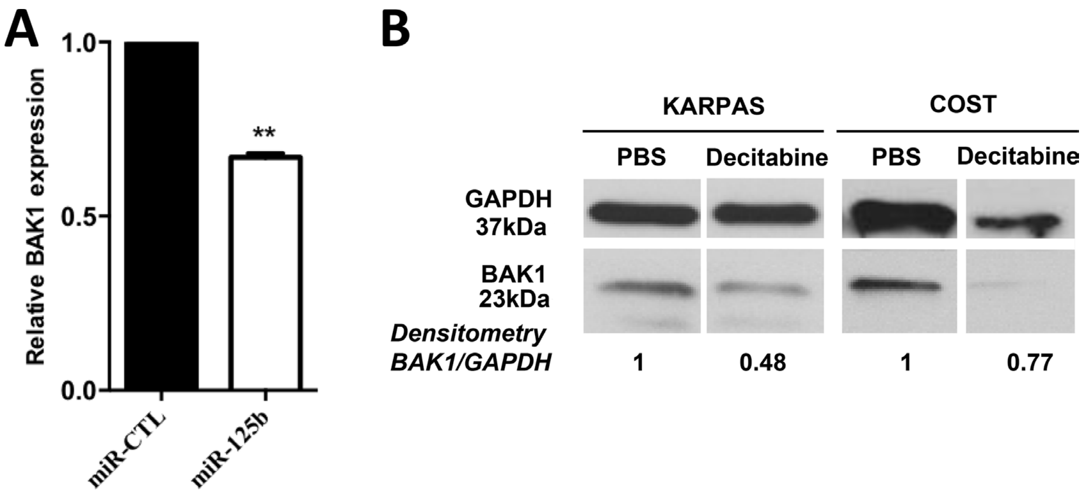

Supplementary Figure 3: MiR-125b targets BAK1 mRNA in NPM-ALK-positive ALCL cells. (**A**) Quantitative RT-PCR analysis of BAK1 expression in KARPAS-299 cells transfected with mimic miR-125b (miR-125b) or miR-CTL (negative control). GAPDH was used as an internal control and the relative BAK1 expression was expressed as $2^{-\Delta\Delta Ct}$ relative to cells transfected with miR-CTL. (**B**) Western blotting analysis of BAK1 and GAPDH expression in KARPAS-299 and COST cells treated or not (PBS) with decitabine. The relative levels of the proteins of interest were normalized to GAPDH levels. Densitometric analysis was performed using GeneTools software from Syngene. Data represent means ± SEM (bars) from 3 independent experiments. $^{**}P < 0.001$; unpaired 2-tailed Student's *t* test.

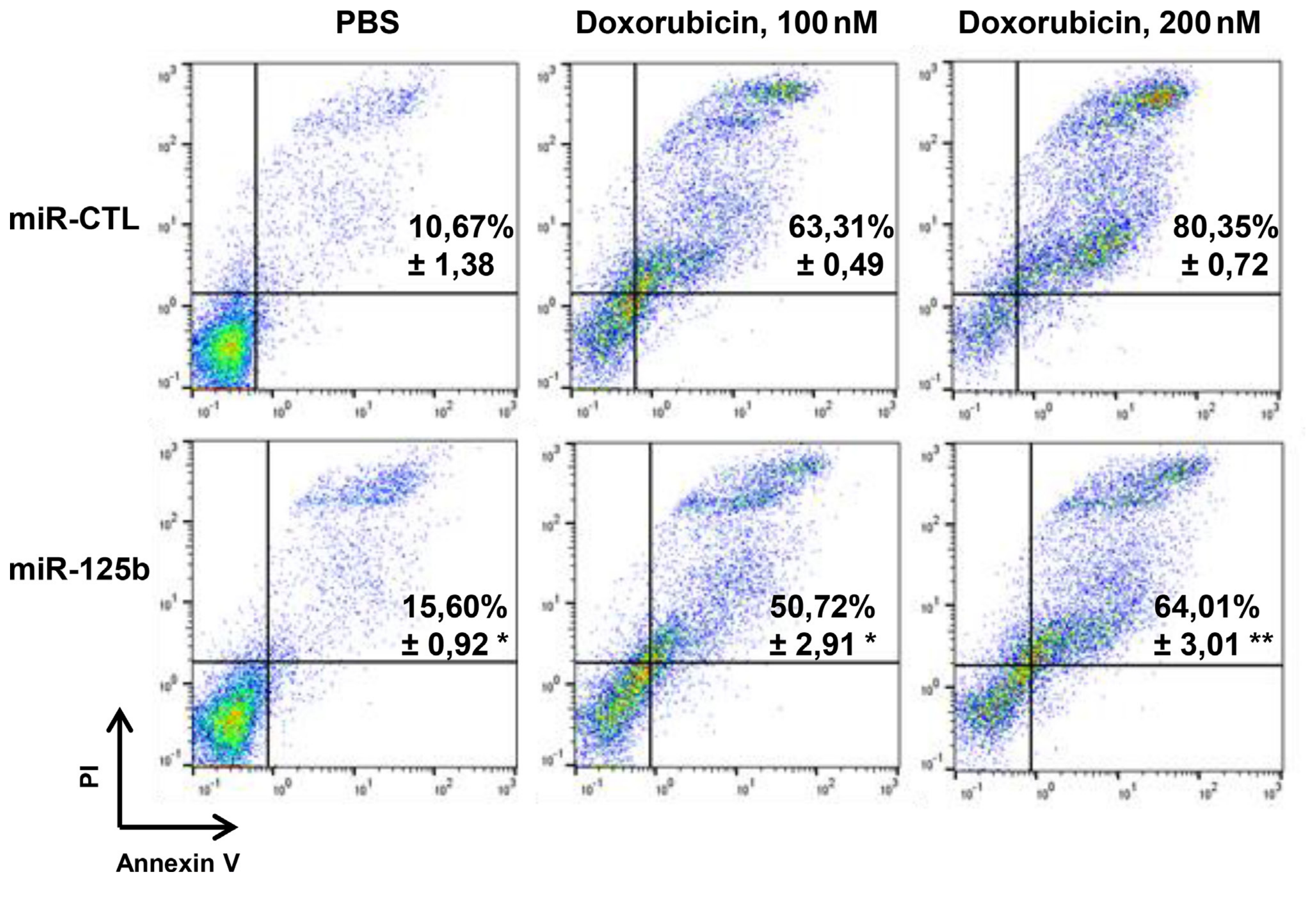

Supplementary Figure 4: Evaluation of apoptosis by Annexin V-pacific blue/IP staining, followed by flow cytometry analysis. Representative plots of Annexin V-pacific blue/IP staining of NPM-ALK(+) ALCL COST cells transfected with mimic miR-125b (miR-125b) or negative control microRNA (miR-CTL) and treated with 0, 100 or 200 nM of doxorubicin. Data showing the percentage of apoptotic cells (Annexin V-pacific blue positive cells) are the mean ± SEM. of three different experiments, each performed in duplicate. $^{*}P < 0.05$ and $^{**}P < 0.01$ using unpaired 2-tailed Student's t test for the comparison between untreated and doxorubicin -treated cells.

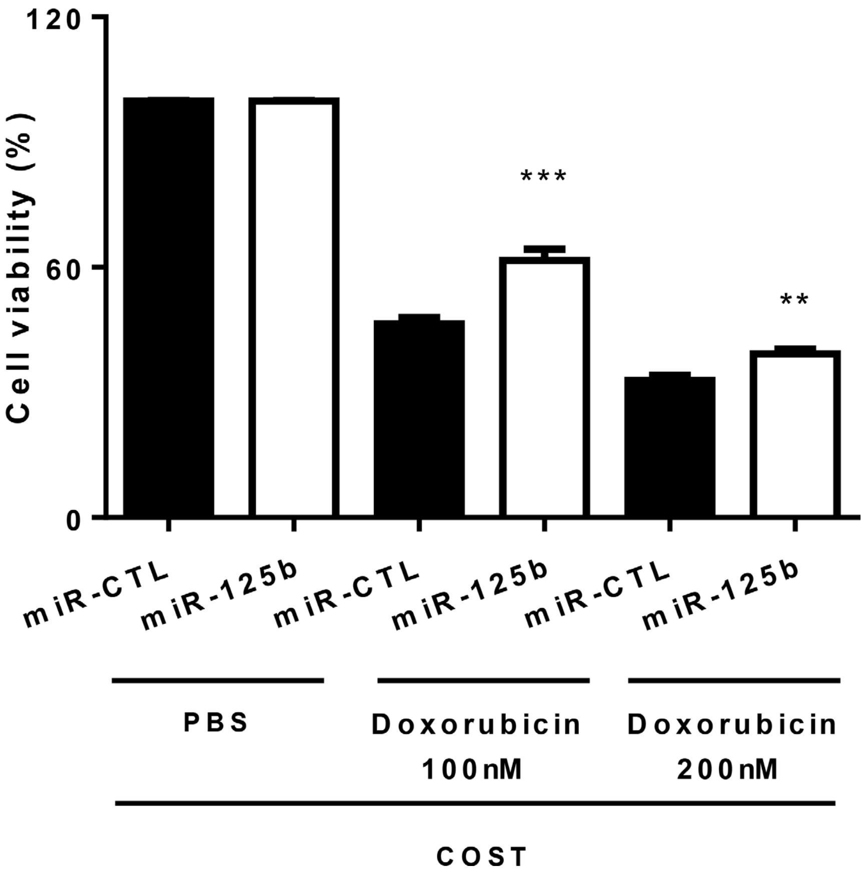

Supplementary Figure 5: Cell proliferation upon doxorubicin treatment in NPM-ALK-positive ALCL cells overexpressing miR-125b. MTS assay of NPM-ALK(+) ALCL COST cells transfected with mimic miR-125b (miR-125b) or negative control microRNA (miR-CTL) and treated with 0, 100 and 200 nM of doxorubicin. Cell growth is expressed as a percentage of the number of untreated cells under the same conditions (PBS). Data represent means ± SEM (bars) from three independent biological replicates $^{**}P < 0.001$ and $^{***}P < 0.0001$; unpaired 2-tailed Student's *t* test.

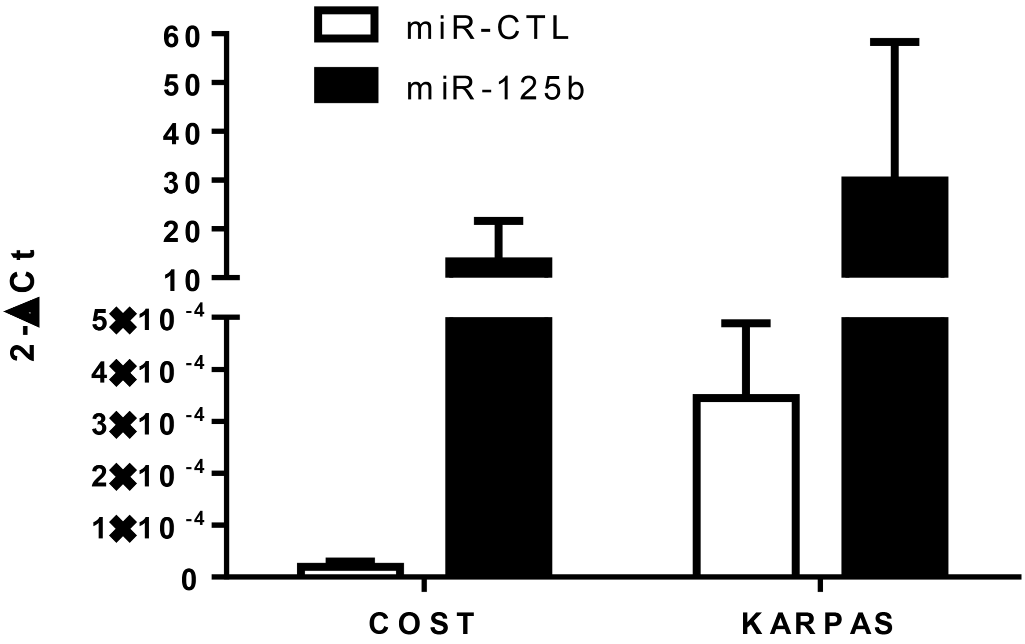

Supplementary Figure 6: Efficiency of miR-125b mimic transfection in COST and KARPAS-299 cells. Cells were transfected with control microRNA (miR-CTL) or miR-125b microRNA (miR-125b) and miR-125b expression was analyzed 72h after transfection by qRT-PCR. SNORD44 served as a relative control. The relative expression levels of miR-125b were expressed as the $2^{-\Delta Ct}$.

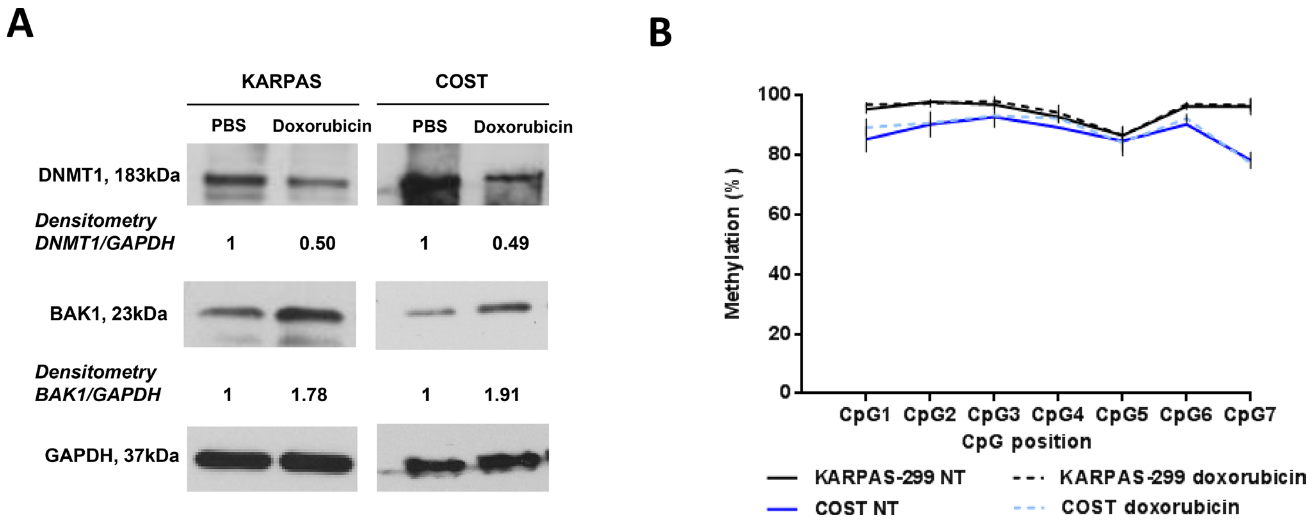

Supplementary Figure 7: Doxorubicin treatment affects the expression of DNMT1 and BAK1. (**A**) Western blotting analysis of DNMT1, BAK1 and GAPDH proteins and in KARPAS-299 and COST cells treated with doxorubicin or vehicle (PBS) (**B**) percentage of DNA methylation, assessed by bisulfite conversion and pyrosequencing in KARPAS-299 (KARPAS) and COST cells treated or not (PBS or NT) with doxorubicin. Data represent means ± SEM (bars) from 3 independent experiments.

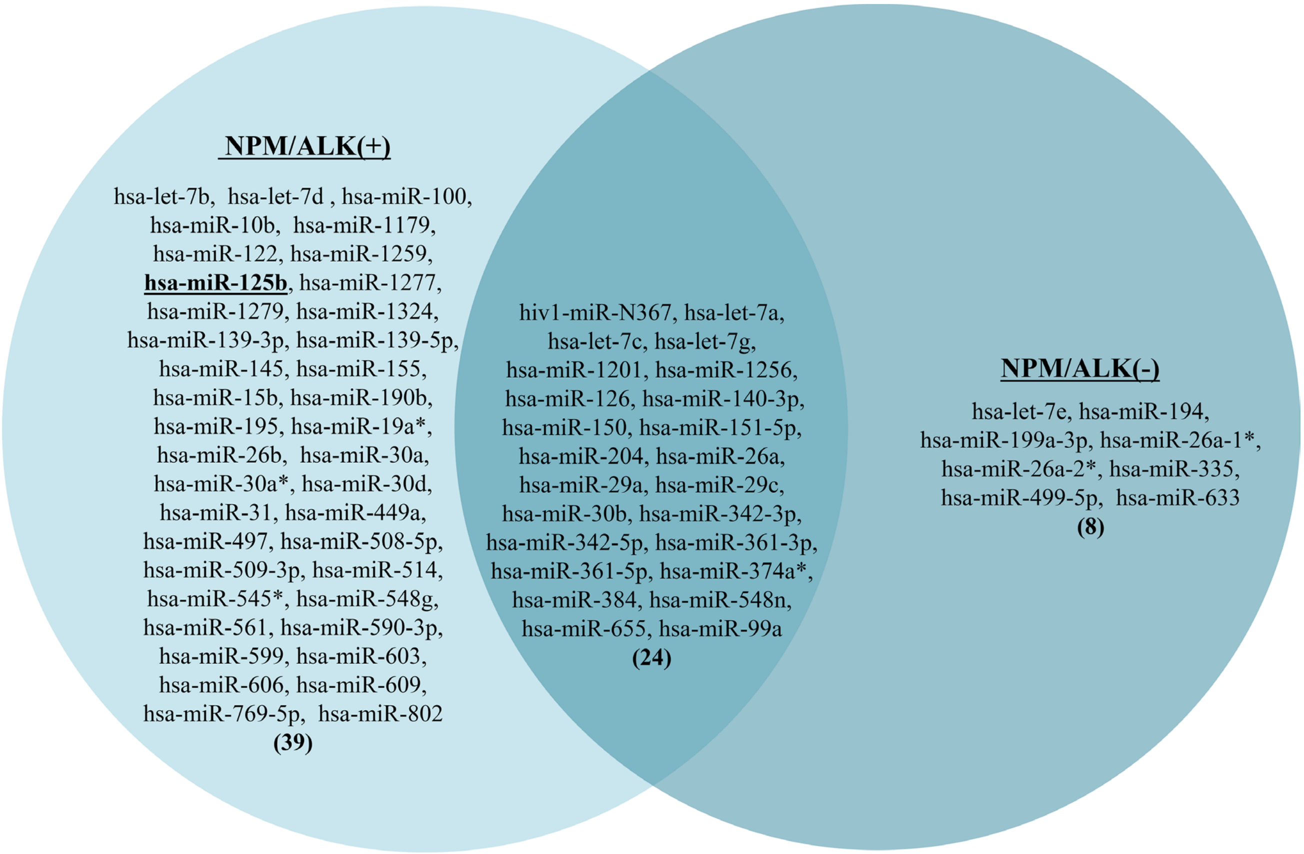

Supplementary Figure 8: Venn diagram of downregulated miRNA analysis in ALCL primary biopsies. Venn diagram used to identify overlapping and non-overlapping downregulated miRNAs in the analysis of Agilent microarrays, which included 866 human and 89 human viral miRNA probes. 52 NPM-ALK(+) and 5 NPM-ALK(-) ALCL primary tissues obtained at the time of diagnosis were used for microarray analysis. Three reactive lymph node tissue samples were used for tissue comparison. The numbers indicate the number of miRNAs in each indicated area.

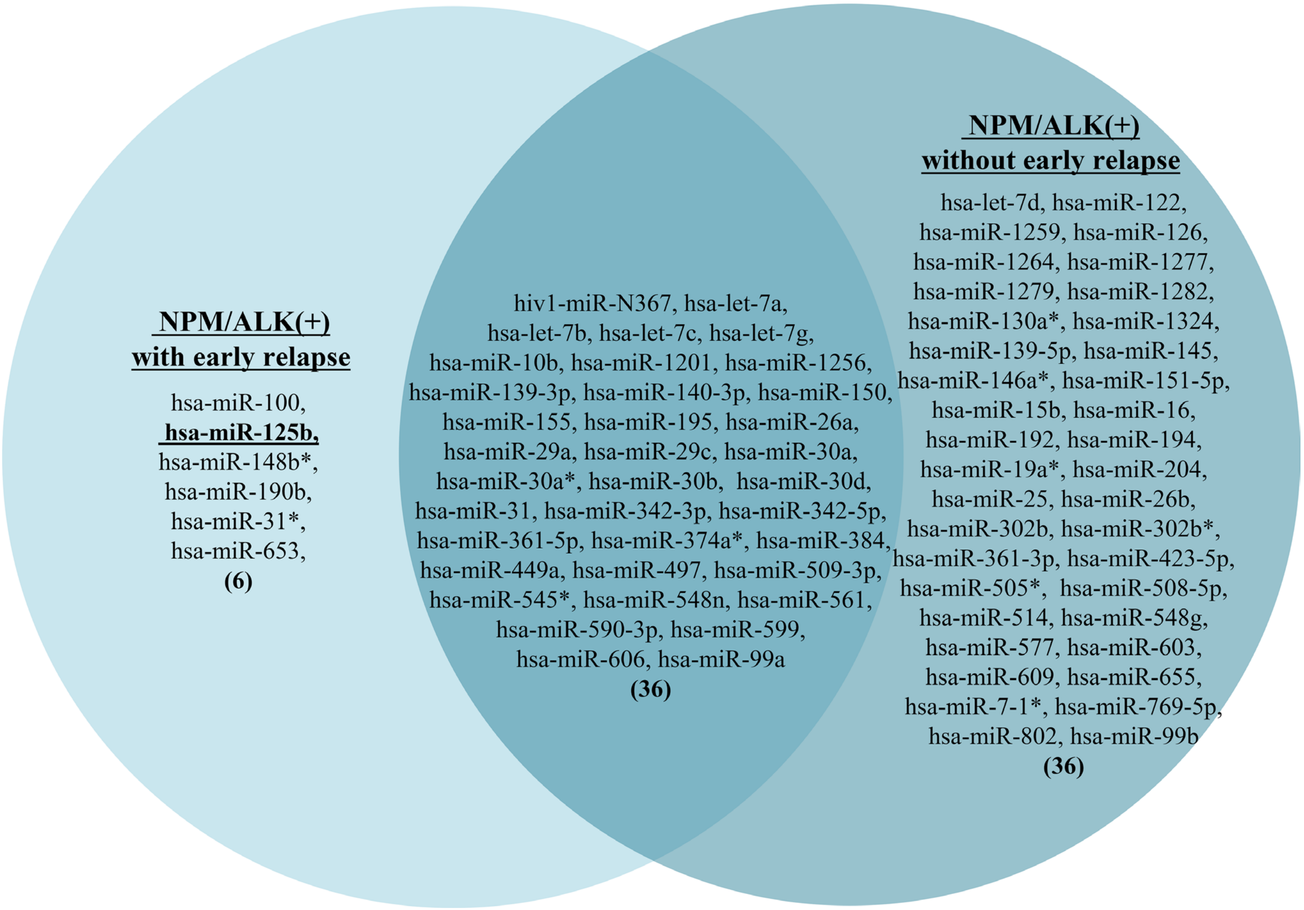

Supplementary Figure 9: Venn diagram of downregulated miRNA analysis in NPM-ALK-positive ALCL primary biopsies from patients who experienced an early relapse. Venn diagram used to identify overlapping and non-overlapping downregulated miRNAs in the analysis of Agilent microarrays which included 866 human and 89 human viral miRNA probes. NPM-ALK(+) ALCL primary biopsies from early relapsing (with, *n* = 28) and non-relapsing (without, *n* = 24) patients after 3 years of minimal follow-up were used for microarray analysis. Three reactive lymph node tissue samples were used for tissue comparison. The numbers indicate the number of miRNAs in each indicated area.

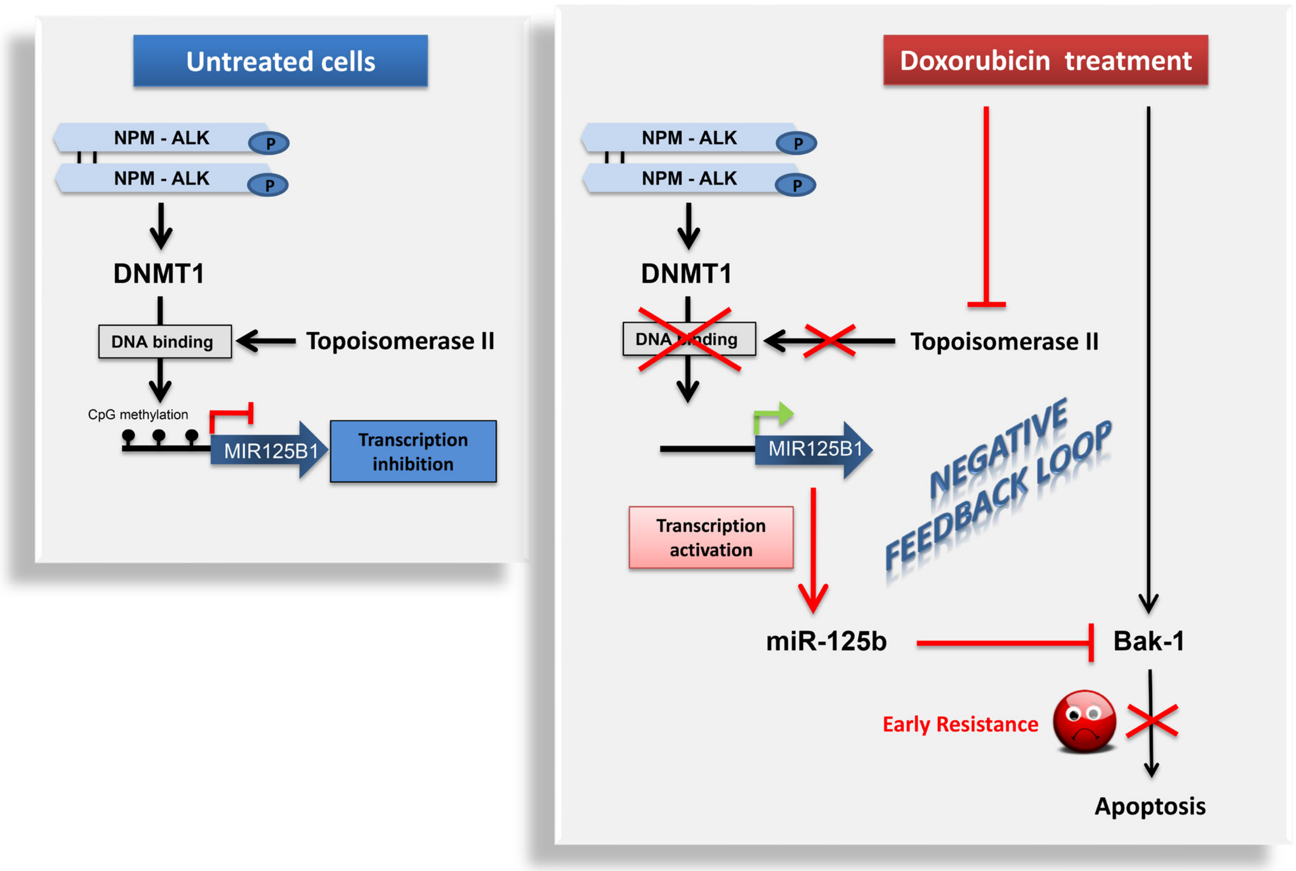

Supplementary Figure 10: Schematic model of the NPM-ALK/DNMT1/DNA topoisomerase II/miR-125b/BAK1 regulatory circuit in NPM-ALK-positive lymphomas. In chemotherapy-naïve anaplastic lymphoma cells, the NPM-ALK fusion protein cooperates with DNA methyltransferase 1 (DNMT1) and DNA topoisomerase II (Topoisomerase II), a DNA-binding protein that regulates DNA methylation, to repress miR-125b expression through DNA hypermethylation. The anti-cancer chemotherapy drug doxorubicin inhibits Topoisomerase II, markedly reducing DNMT1 binding to the *MIR125B1* promoter and releasing miR-125b inhibition. Increased miR-125b expression then leads to resistance against doxorubicin via the repression of the pro-apoptotic protein BAK1 (BCL2 antagonist/killer 1), a bona fide miR-125b target.

Supplementary Table 1: Sequences of si-RNAs and primers used for quantitative real-time PCR, pyrosequencing and chromatin immunoprecipitation analysis

**Genes**	**Sequence of RT-qPCR primers (5'-3')**
hsa-BAK1-F	CAACCGACGCTATGACTC
hsa-BAK1-R	AATTGATGCCACTCTCAAAC
hsa-PUMA-F	CGGGGAGGAGGAACAGTGGG
hsa-PUMA-R	CCAGGGTGAGGGGCGGTG
hsa-P53-F	ACTAAGCGAGCACTGCCCAAC
hsa-P53-R	GCTCACGCCCACGGATCTGA
hsa-MCL1-F	AGCTGCATCGAACCATTAGC
hsa-MCL1-R	TCCTGATGCCACCTTCTAGG
hsa-GAPDH-F	CGGGAAGCTTGTGATCAATGG
hsa-GAPDH-R	GGCAGTGATGGCATGGACTG
hsa-NPM-ALK-F	CAGTGCATATTAGTGGACAGCACTTAG
hsa-NPM-ALK-R	TGATGGTCGAGGTGCGGA
**Genes**	**Pyrosequencing primers (5'-3')**
m125_F1	GATGGTGTTATAGGAGGTTGT
m125_Rb1	[Btn]AACATCTCTTTCCCCCAAAAC
m125_S1	TGTTATAGGAGGTTGTG
m125_F2	TGGGTATTTTTGAGTTTGTGTATATTA
m125_Rb2	[Btn]TCTAAACTCCTTTCCTTCACAAAATTA
m125_S2	TTGAAAGAGTTTTTTTTGATG
**Genes**	**ChIP primers (5'-3')**
ChIP-miR125b-F	CAGGAGGCTGTGCGGGC
ChIP-miR125b-R	AGACCAAGAAGTAGGATTCGGAGGC
**Genes**	**siRNA sequences (5'-3')**
NPM-ALK si-RNAs	GGGCGAGCUACUAUAGAAA
STAT3 si-RNAs	AACAUCUGCCUAGAACGGCUA
BAK1 si-RNAs	UGGUACGAAGAUUCUUCAAAC)TT
Si-CTL	Smart Pool 4609 (Dharmacon)

Supplementary Table 2: List of primary antibodies used in western blotting and chromatin immunoprecipitation

**Primary antibodies used for western blotting**		
**Antibodies**	**Clone**	**Source**
NPM-ALK	D5F3, rabbit monoclonal	Cell Signaling Tech
P-NPM-ALK	CS3341, rabbit monoclonal	Cell Signaling Tech
STAT3	CS9132, rabbit monoclonal	Cell Signaling Tech
P-STAT3	CS9131, rabbit polyclonal	Cell Signaling Tech
BAK1	D4E4, rabbit monoclonal	Cell Signaling Tech
GAPDH	MAB374, rabbit monoclonal	Chemicon
**Primary antibodies used for ChIP**		
DNMT1	39204, mouse monoclonal	Active motif
H3K27me3	39535, mouse monoclonal	Active motif

Supplementary Table 3: MiRNAs with differential expression in NPM-ALK(+) ALCL lymph node primary tissues at diagnosis, sorted according to fold change in expression. See_Supplementary Table 3

Supplementary Table 4: MiRNAs with differential expression in NPM-ALK(-) ALCL lymph node primary tissues, sorted according to the fold change in expression

	miRNA_ID	Fold Change	adj.P.Val
	**Upregulated miRNAs in human NPM-ALK(-) ALCL lymph node primary tissues†**		
1	hsa-miR-409-3p	16.517	3.41E-02
2	hsa-miR-21*	*7.992*	*3.82E-02*
3	hsa-miR-21	4.130	3.13E-02
	**Downregulated miRNAs in human NPM-ALK(-) ALCL lymph node primary tissues††**		
1	hsa-miR-199a-3p	0.455	4.17E-02
2	hsa-let-7e	0.365	3.41E-02
3	hsa-miR-361-5p	0.358	3.41E-02
4	hiv1-miR-N367	0.343	3.85E-03
5	hsa-miR-655	0.326	3.82E-02
6	hsa-miR-26a-1*	0.324	3.82E-02
7	hsa-miR-26a-2*	0.302	3.82E-02
8	hsa-miR-548n	0.299	1.43E-02
9	hsa-miR-384	0.294	1.24E-02
10	hsa-miR-633	0.282	4.78E-03
11	hsa-miR-1256	0.252	3.85E-03
12	hsa-let-7a	0.250	2.64E-02
13	hsa-miR-151-5p	0.250	2.47E-02
14	hsa-miR-29a	0.249	1.24E-02
15	hsa-miR-1201	0.215	3.50E-03
16	hsa-miR-26a	0.214	3.82E-02
17	hsa-let-7c	0.214	8.36E-03
18	hsa-miR-29c	0.208	2.64E-02
19	hsa-miR-374a*	0.206	1.78E-02
20	hsa-miR-140-3p	0.205	2.28E-02
21	hsa-miR-342-3p	0.203	2.64E-02
22	hsa-let-7g	0.181	2.18E-02
23	hsa-miR-126	0.175	4.83E-03
24	hsa-miR-361-3p	0.160	2.66E-02
25	hsa-miR-194	0.143	3.41E-02
26	hsa-miR-30b	0.140	1.24E-02
27	hsa-miR-342-5p	0.094	2.64E-02
28	hsa-miR-499-5p	0.083	4.96E-02
29	hsa-miR-99a	0.070	1.24E-02
30	hsa-miR-204	0.047	1.24E-02
31	hsa-miR-150	0.037	1.24E-02
32	hsa-miR-335	0.034	2.18E-02

Intensity of signal was (†) > 1.5 fold and (††) < 0.5 fold compared with that from the lymph nodes of healthy donors (n = 3).

Supplementary Table 5: MiRNAs with differential expression in NPM-ALK(+) ALCL lymph node primary tissues from patients who experienced an early relapse, sorted according to the fold change in expression. See_Supplementary Table 5

Supplementary Table 6: MiRNAs with differential expression in NPM-ALK(+) ALCL lymph node primary tissues from patients without relapse after 3 years of minimal follow-up, sorted according to the fold change in expression. See_Supplementary Table 6
