# Supplementary material for: Doxorubicin-induced loss of DNA topoisomerase II and DNMT1- dependent suppression of MiR-125b induces chemoresistance in ALK-positive cells
Source: Oncotarget. 2018 Feb 8;9(18):14539–51. doi: 10.18632/oncotarget.24465 (PMC5865688; doi:10.18632/oncotarget.24465)
Supplement: Supplementary file 2 [file oncotarget-09-14539-s002.docx]

**Supplementary Table 3:** **MiRNAs with differential expression in NPM-ALK(+) ALCL lymph node primary tissues at diagnosis, sorted according to fold change in expression**

| **Upregulated miRNAs in human NPM-ALK(+) ALCL lymph node primary tissues†** | | | |
| --- | --- | --- | --- |
|  | **miRNA_ID** | **Fold Change** | **adj.P.Val** |
|  | hsa-miR-135b | 28.452 | 1.19E-05 |
|  | hsa-miR-503 | 14.412 | 9.33E-03 |
|  | hsa-miR-409-3p | 12.385 | 1.64E-04 |
|  | hsa-miR-21* | 9.574 | 7.05E-11 |
|  | hsa-miR-154* | 6.333 | 2.85E-03 |
|  | hsa-miR-708 | 5.660 | 3.58E-02 |
|  | hsa-miR-21 | 4.893 | 7.05E-11 |
|  | hsa-miR-542-3p | 4.513 | 1.95E-02 |
|  | hsa-miR-634 | 4.336 | 2.19E-02 |
|  | hsa-miR-424 | 3.697 | 2.22E-02 |
|  | hsa-miR-421 | 3.315 | 2.83E-02 |
|  | hsa-miR-602 | 3.171 | 3.29E-02 |
|  | hsa-miR-1274a | 2.893 | 1.85E-03 |
|  | hsa-miR-1280 | 2.864 | 5.54E-04 |
|  | hsa-miR-487b | 2.664 | 7.16E-03 |
|  | hsa-miR-550* | 2.499 | 2.76E-02 |
|  | hsa-miR-301a | 2.159 | 1.34E-02 |
|  | hsa-miR-1274b | 1.944 | 5.89E-03 |
|  | **Downregulated miRNAs in human NPM-ALK(+) ALCL lymph node primary tissues††** | | |
|  | hsa-miR-545* | 0.647 | 4.27E-04 |
|  | hsa-let-7d | 0.645 | 4.43E-02 |
|  | hsa-miR-561 | 0.633 | 1.08E-03 |
|  | hsa-miR-15b | 0.630 | 3.29E-02 |
|  | hsa-miR-802 | 0.628 | 1.72E-02 |
|  | hsa-miR-19a* | 0.609 | 3.93E-02 |
|  | hsa-miR-509-3p | 0.583 | 1.28E-03 |
|  | hsa-miR-361-5p | 0.561 | 2.40E-03 |
|  | hsa-miR-30d | 0.551 | 2.85E-03 |
|  | hsa-miR-599 | 0.546 | 1.08E-03 |
|  | hsa-miR-603 | 0.540 | 2.74E-02 |
|  | hsa-miR-1179 | 0.537 | 4.27E-02 |
|  | hsa-miR-190b | 0.491 | 3.14E-02 |
|  | hsa-let-7a | 0.484 | 1.00E-03 |
|  | hsa-miR-606 | 0.461 | 4.09E-04 |
|  | hsa-miR-769-5p | 0.460 | 4.43E-02 |
|  | hsa-miR-126 | 0.432 | 1.34E-02 |
|  | hsa-miR-151-5p | 0.428 | 1.18E-02 |
|  | hsa-miR-26b | 0.424 | 2.98E-02 |
|  | hsa-miR-122 | 0.421 | 3.71E-02 |
|  | hsa-miR-29c | 0.408 | 6.26E-04 |
|  | hsa-let-7g | 0.407 | 9.66E-04 |
|  | hsa-miR-1324 | 0.405 | 1.28E-02 |
|  | hsa-miR-1279 | 0.395 | 3.93E-02 |
|  | hsa-let-7c | 0.378 | 2.62E-03 |
|  | hsa-miR-139-3p | 0.368 | 8.56E-03 |
|  | hiv1-miR-N367 | 0.362 | 1.31E-07 |
|  | hsa-miR-29a | 0.360 | 4.64E-07 |
|  | hsa-miR-1277 | 0.354 | 2.83E-02 |
|  | hsa-miR-514 | 0.353 | 1.85E-03 |
|  | hsa-miR-655 | 0.351 | 7.15E-03 |
|  | hsa-miR-384 | 0.349 | 5.54E-04 |
|  | hsa-let-7b | 0.349 | 2.85E-03 |
|  | hsa-miR-361-3p | 0.339 | 4.35E-02 |
|  | hsa-miR-1259 | 0.332 | 8.81E-03 |
|  | hsa-miR-140-3p | 0.332 | 6.22E-03 |
|  | hsa-miR-548n | 0.325 | 7.39E-06 |
|  | hsa-miR-30b | 0.325 | 1.85E-03 |
|  | hsa-miR-609 | 0.321 | 2.85E-03 |
|  | hsa-miR-342-3p | 0.314 | 3.13E-03 |
|  | hsa-miR-30a | 0.311 | 5.39E-03 |
|  | hsa-miR-10b | 0.301 | 1.08E-03 |
|  | hsa-miR-125b | 0.300 | 2.27E-02 |
|  | hsa-miR-195 | 0.298 | 8.56E-03 |
|  | hsa-miR-590-3p | 0.297 | 4.09E-04 |
|  | hsa-miR-1256 | 0.290 | 1.85E-03 |
|  | hsa-miR-1201 | 0.288 | 1.30E-02 |
|  | hsa-miR-26a | 0.280 | 2.85E-03 |
|  | hsa-miR-497 | 0.262 | 2.22E-03 |
|  | hsa-miR-508-5p | 0.255 | 1.18E-02 |
|  | hsa-miR-548g | 0.254 | 2.82E-02 |
|  | hsa-miR-145 | 0.242 | 3.29E-02 |
|  | hsa-miR-374a* | 0.230 | 5.07E-05 |
|  | hsa-miR-155 | 0.201 | 4.39E-03 |
|  | hsa-miR-100 | 0.193 | 2.55E-02 |
|  | hsa-miR-342-5p | 0.170 | 5.39E-03 |
|  | hsa-miR-139-5p | 0.159 | 3.93E-02 |
|  | hsa-miR-30a* | 0.144 | 1.76E-02 |
|  | hsa-miR-204 | 0.137 | 2.58E-02 |
|  | hsa-miR-99a | 0.125 | 3.68E-03 |
|  | hsa-miR-449a | 0.120 | 2.60E-03 |
|  | hsa-miR-150 | 0.093 | 3.68E-03 |
|  | hsa-miR-31 | 0.060 | 1.58E-02 |

Intensity of signal was (†) > 1.5 fold and (††) < 0.5 fold compared with that from the lymph nodes of healthy donors (n=3).
