# Supplementary material for: Doxorubicin-induced loss of DNA topoisomerase II and DNMT1- dependent suppression of MiR-125b induces chemoresistance in ALK-positive cells
Source: Oncotarget. 2018 Feb 8;9(18):14539–51. doi: 10.18632/oncotarget.24465 (PMC5865688; doi:10.18632/oncotarget.24465)
Supplement: Supplementary file 3 [file oncotarget-09-14539-s003.docx]

**Supplementary Table 5: MiRNAs with differential expression in NPM-ALK(+) ALCL lymph node primary tissues from patients who experienced an early relapse, sorted according to the fold change in expression**

|  | **Upregulated miRNAs in NPM-ALK(+) ALCL lymph node primary tissues from patients who experienced an early relapse†** | | |
| --- | --- | --- | --- |
|  | **miRNA_ID** | **Fold Change** | **adj.P.Val** |
|  | hsa-miR-135b | 23.398 | 1.73E-04 |
|  | hsa-miR-21* | 10.340 | 1.57E-06 |
|  | hsa-miR-409-3p | 9.619 | 7.50E-03 |
|  | hsa-miR-99b* | 6.314 | 2.41E-02 |
|  | hsa-miR-154* | 6.182 | 1.35E-02 |
|  | hsa-miR-21 | 5.018 | 1.57E-06 |
|  | hsa-miR-634 | 3.894 | 2.74E-02 |
|  | hsa-miR-424 | 3.569 | 4.31E-02 |
|  | hsa-miR-1274a | 3.189 | 7.32E-03 |
|  | hsa-miR-1280 | 2.980 | 6.33E-03 |
|  | hsa-miR-602 | 2.870 | 4.80E-02 |
|  | hsa-miR-487b | 2.864 | 1.94E-03 |
|  | hsa-miR-550* | 2.745 | 2.74E-02 |
|  | hsa-miR-34a | 2.349 | 2.41E-02 |
|  | hsa-miR-212 | 2.284 | 4.25E-02 |
|  | hsa-miR-301a | 2.218 | 2.33E-02 |
|  | hsa-miR-1274b | 2.191 | 1.21E-02 |
|  | **Downregulated miRNAs in NPM-ALK(+) ALCL lymph node primary tissues from patients who experienced an early relapse††** | | |
|  | hsa-miR-653 | 0.684 | 4.80E-02 |
|  | hsa-miR-545* | 0.649 | 1.20E-02 |
|  | hsa-miR-561 | 0.633 | 3.71E-02 |
|  | hsa-miR-361-5p | 0.593 | 9.76E-03 |
|  | hsa-miR-509-3p | 0.586 | 4.25E-02 |
|  | hsa-miR-30d | 0.572 | 1.02E-02 |
|  | hsa-miR-599 | 0.546 | 3.71E-02 |
|  | hsa-let-7a | 0.544 | 6.34E-03 |
|  | hsa-let-7g | 0.480 | 2.06E-03 |
|  | hsa-miR-190b | 0.459 | 1.21E-02 |
|  | hsa-miR-29c | 0.453 | 2.06E-03 |
|  | hsa-miR-606 | 0.453 | 2.76E-03 |
|  | hsa-miR-140-3p | 0.405 | 9.76E-03 |
|  | hsa-miR-148b* | 0.404 | 4.88E-02 |
|  | hsa-let-7c | 0.403 | 1.21E-02 |
|  | hsa-miR-29a | 0.397 | 3.42E-05 |
|  | hsa-let-7b | 0.379 | 2.41E-02 |
|  | hsa-miR-342-3p | 0.373 | 6.33E-03 |
|  | hiv1-miR-N367 | 0.371 | 5.32E-04 |
|  | hsa-miR-30b | 0.369 | 7.50E-03 |
|  | hsa-miR-139-3p | 0.368 | 3.48E-02 |
|  | hsa-miR-30a | 0.358 | 2.33E-02 |
|  | hsa-miR-384 | 0.342 | 1.74E-03 |
|  | hsa-miR-195 | 0.335 | 4.88E-02 |
|  | hsa-miR-10b | 0.320 | 8.56E-03 |
|  | hsa-miR-548n | 0.312 | 4.00E-06 |
|  | hsa-miR-590-3p | 0.303 | 2.76E-03 |
|  | hsa-miR-125b | 0.303 | 2.41E-02 |
|  | hsa-miR-26a | 0.300 | 1.21E-02 |
|  | hsa-miR-497 | 0.289 | 1.60E-02 |
|  | hsa-miR-1201 | 0.280 | 2.31E-02 |
|  | hsa-miR-1256 | 0.278 | 6.33E-03 |
|  | hsa-miR-374a* | 0.246 | 8.23E-03 |
|  | hsa-miR-342-5p | 0.225 | 2.76E-03 |
|  | hsa-miR-100 | 0.216 | 3.35E-02 |
|  | hsa-miR-155 | 0.177 | 3.18E-03 |
|  | hsa-miR-30a* | 0.176 | 3.35E-02 |
|  | hsa-miR-31* | 0.147 | 4.26E-02 |
|  | hsa-miR-99a | 0.131 | 1.39E-02 |
|  | hsa-miR-449a | 0.131 | 1.60E-02 |
|  | hsa-miR-150 | 0.129 | 6.62E-03 |
|  | hsa-miR-31 | 0.055 | 1.60E-02 |

Intensity of signal was (**†**) > 1.5 fold and (**††**) < 0.5 fold compared with that from the lymph nodes of healthy donors (n=3).
