# Supplementary material for: Doxorubicin-induced loss of DNA topoisomerase II and DNMT1- dependent suppression of MiR-125b induces chemoresistance in ALK-positive cells
Source: Oncotarget. 2018 Feb 8;9(18):14539–51. doi: 10.18632/oncotarget.24465 (PMC5865688; doi:10.18632/oncotarget.24465)
Supplement: Supplementary file 4 [file oncotarget-09-14539-s004.docx]

**Supplementary Table 6: MiRNAs with differential expression in NPM-ALK(+) ALCL lymph node primary tissues from patients without relapse after 3 years of minimal follow-up, sorted according to the fold change in expression**

|  | **Upregulated miRNAs in NPM-ALK(+) ALCL lymph node primary tissues from patients without relapse †** | | |
| --- | --- | --- | --- |
|  | **miRNA_ID** | **Fold Change** | **adj.P.Val** |
|  | hsa-miR-135b | 35.725 | 1.12E-04 |
|  | hsa-miR-409-3p | 16.670 | 3.19E-06 |
|  | hsa-miR-503 | 16.395 | 3.37E-03 |
|  | hsa-miR-21* | 8.709 | 3.19E-06 |
|  | hsa-miR-154* | 6.503 | 8.59E-03 |
|  | hsa-miR-636 | 6.416 | 4.70E-02 |
|  | hsa-miR-708 | 6.339 | 3.37E-03 |
|  | hsa-miR-379 | 5.790 | 1.81E-02 |
|  | hsa-miR-542-3p | 5.055 | 3.37E-03 |
|  | hsa-miR-631 | 4.790 | 4.84E-02 |
|  | hsa-miR-21 | 4.721 | 1.87E-08 |
|  | hsa-miR-886-3p | 4.385 | 4.70E-02 |
|  | hsa-miR-424 | 3.851 | 3.39E-02 |
|  | hsa-miR-602 | 3.559 | 4.70E-02 |
|  | hsa-miR-421 | 3.538 | 2.22E-02 |
|  | hsa-miR-377 | 3.290 | 4.67E-02 |
|  | hsa-miR-376c | 3.195 | 4.95E-02 |
|  | hsa-miR-1280 | 2.709 | 4.29E-03 |
|  | hsa-miR-1274a | 2.568 | 2.18E-02 |
|  | **Downregulated miRNAs in NPM-ALK(+) ALCL lymph node primary tissues from patients without relapse ††** | | |
|  | hsa-miR-302b* | 0.660 | 4.24E-03 |
|  | hsa-miR-25 | 0.650 | 3.79E-02 |
|  | hsa-miR-545* | 0.645 | 5.93E-03 |
|  | hsa-miR-561 | 0.632 | 4.70E-02 |
|  | hsa-miR-19a* | 0.604 | 3.30E-02 |
|  | hsa-miR-802 | 0.601 | 5.01E-04 |
|  | hsa-miR-130a* | 0.579 | 4.70E-02 |
|  | hsa-miR-509-3p | 0.579 | 4.70E-02 |
|  | hsa-miR-15b | 0.568 | 1.87E-02 |
|  | hsa-let-7d | 0.567 | 2.30E-02 |
|  | hsa-miR-16 | 0.563 | 2.51E-02 |
|  | hsa-miR-599 | 0.545 | 4.70E-02 |
|  | hsa-miR-577 | 0.542 | 5.01E-04 |
|  | hsa-miR-30d | 0.527 | 7.80E-03 |
|  | hsa-miR-361-5p | 0.523 | 9.42E-03 |
|  | hsa-miR-603 | 0.519 | 4.82E-02 |
|  | hsa-miR-99b | 0.488 | 3.96E-02 |
|  | hsa-miR-606 | 0.469 | 1.14E-02 |
|  | hsa-miR-769-5p | 0.456 | 2.34E-02 |
|  | hsa-let-7a | 0.419 | 4.34E-03 |
|  | hsa-miR-7-1* | 0.408 | 1.17E-02 |
|  | hsa-miR-151-5p | 0.384 | 3.37E-03 |
|  | hsa-miR-122 | 0.377 | 7.80E-03 |
|  | hsa-miR-126 | 0.373 | 3.37E-03 |
|  | hsa-miR-423-5p | 0.371 | 6.44E-03 |
|  | hsa-miR-146a* | 0.369 | 4.83E-02 |
|  | hsa-miR-1264 | 0.368 | 6.27E-03 |
|  | hsa-miR-139-3p | 0.368 | 7.80E-03 |
|  | hsa-miR-1279 | 0.366 | 4.29E-03 |
|  | hsa-miR-1324 | 0.358 | 4.64E-06 |
|  | hsa-miR-29c | 0.358 | 3.37E-03 |
|  | hsa-miR-384 | 0.357 | 6.27E-03 |
|  | hsa-miR-26b | 0.354 | 2.85E-02 |
|  | hiv1-miR-N367 | 0.352 | 1.87E-08 |
|  | hsa-let-7c | 0.350 | 6.85E-03 |
|  | hsa-miR-548n | 0.340 | 3.09E-03 |
|  | hsa-miR-514 | 0.340 | 5.78E-03 |
|  | hsa-let-7g | 0.336 | 2.88E-03 |
|  | hsa-miR-1282 | 0.335 | 7.51E-03 |
|  | hsa-miR-1277 | 0.333 | 3.79E-02 |
|  | hsa-miR-655 | 0.324 | 2.58E-04 |
|  | hsa-miR-29a | 0.319 | 4.08E-05 |
|  | hsa-let-7b | 0.314 | 4.24E-03 |
|  | hsa-miR-1256 | 0.303 | 6.85E-03 |
|  | hsa-miR-1259 | 0.301 | 3.37E-03 |
|  | hsa-miR-1201 | 0.296 | 3.83E-02 |
|  | hsa-miR-609 | 0.296 | 3.37E-03 |
|  | hsa-miR-192 | 0.293 | 4.82E-02 |
|  | hsa-miR-590-3p | 0.290 | 3.69E-03 |
|  | hsa-miR-302b | 0.287 | 3.30E-02 |
|  | hsa-miR-361-3p | 0.284 | 4.70E-02 |
|  | hsa-miR-10b | 0.279 | 3.37E-03 |
|  | hsa-miR-30b | 0.278 | 3.99E-03 |
|  | hsa-miR-505* | 0.266 | 4.82E-02 |
|  | hsa-miR-30a | 0.264 | 6.27E-03 |
|  | hsa-miR-140-3p | 0.263 | 7.20E-03 |
|  | hsa-miR-195 | 0.260 | 6.27E-03 |
|  | hsa-miR-26a | 0.257 | 6.41E-03 |
|  | hsa-miR-342-3p | 0.256 | 6.85E-03 |
|  | hsa-miR-145 | 0.236 | 4.82E-02 |
|  | hsa-miR-497 | 0.235 | 3.37E-03 |
|  | hsa-miR-155 | 0.231 | 3.79E-02 |
|  | hsa-miR-194 | 0.231 | 3.39E-02 |
|  | hsa-miR-508-5p | 0.231 | 6.52E-04 |
|  | hsa-miR-548g | 0.214 | 7.80E-03 |
|  | hsa-miR-374a* | 0.214 | 6.81E-07 |
|  | hsa-miR-139-5p | 0.124 | 1.87E-02 |
|  | hsa-miR-342-5p | 0.122 | 1.14E-02 |
|  | hsa-miR-99a | 0.118 | 8.30E-03 |
|  | hsa-miR-30a* | 0.113 | 2.69E-02 |
|  | hsa-miR-449a | 0.109 | 6.27E-03 |
|  | hsa-miR-204 | 0.107 | 3.37E-03 |
|  | hsa-miR-31 | 0.065 | 4.82E-02 |
|  | hsa-miR-150 | 0.063 | 7.47E-03 |

Intensity of signal was (**†**) > 1.5 fold and (**††**) < 0.5 fold compared with that from the lymph nodes of healthy donors (n=3).
